# Tannins Possessing Bacteriostatic Effect Impair *Pseudomonas aeruginosa* Adhesion and Biofilm Formation

**DOI:** 10.1371/journal.pone.0066257

**Published:** 2013-06-11

**Authors:** Danielle S. Trentin, Denise B. Silva, Matheus W. Amaral, Karine R. Zimmer, Márcia V. Silva, Norberto P. Lopes, Raquel B. Giordani, Alexandre J. Macedo

**Affiliations:** 1 Faculdade de Farmácia, Universidade Federal do Rio Grande do Sul, Porto Alegre, Rio Grande do Sul, Brazil; 2 Centro de Biotecnologia do Estado do Rio Grande do Sul, Porto Alegre, Universidade Federal do Rio Grande do Sul, Porto Alegre, Rio Grande do Sul, Brazil; 3 Núcleo de Pesquisas em Produtos Naturais e Sintéticos (NPPNS), Faculdade de Ciências Farmacêuticas de Ribeirão Preto, Universidade de São Paulo, Ribeirão Preto, São Paulo, Brazil; 4 Lychnoflora Pesquisa e Desenvolvimento em Produtos Naturais LTDA, Incubadora Supera, Campus da Universidade de São Paulo, Ribeirão Preto, São Paulo, Brazil; 5 Centro de Ciências Biológicas e Departamento de Bioquímica, Universidade Federal de Pernambuco, Recife, Pernambuco, Brazil; 6 Centro de Ciências da Saúde, Departamento de Farmácia, Universidade Federal do Rio Grande do Norte, Natal, Rio Grande do Norte, Brazil; LAAS-CNRS, France

## Abstract

Plants produce many compounds that are biologically active, either as part of their normal program of growth and development or in response to pathogen attack or stress. Traditionally, *Anadenanthera colubrina*, *Commiphora leptophloeos* and *Myracrodruon urundeuva* have been used by communities in the Brazilian Caatinga to treat several infectious diseases. The ability to impair bacterial adhesion represents an ideal strategy to combat bacterial pathogenesis, because of its importance in the early stages of the infectious process; thus, the search for anti-adherent compounds in plants is a very promising alternative. This study investigated the ability of stem-bark extracts from these three species to control the growth and prevent biofilm formation of *Pseudomonas aeruginosa*, an important opportunistic pathogen that adheres to surfaces and forms protective biofilms. A kinetic study (0–72 h) demonstrated that the growth of extract-treated bacteria was inhibited up to 9 h after incubation, suggesting a bacteriostatic activity. Transmission electron microscopy and fluorescence microscopy showed both viable and nonviable cells, indicating bacterial membrane damage; crystal violet assay and scanning electron microscopy demonstrated that treatment strongly inhibited biofilm formation during 6 and 24 h and that matrix production remained impaired even after growth was restored, at 24 and 48 h of incubation. Herein, we propose that the identified (condensed and hydrolyzable) tannins are able to inhibit biofilm formation via bacteriostatic properties, damaging the bacterial membrane and hindering matrix production. Our findings demonstrate the importance of this abundant class of Natural Products in higher plants against one of the most challenging issues in the hospital setting: biofilm resilience.

## Introduction

Adhesion and colonization are prerequisites for the establishment of bacterial infection and pathogenesis. Once adhesion has taken place, on implanted medical devices or damaged tissue, microorganisms may undergo specific molecular changes to become pathogenic and to establish biofilms [1]. It is well known that biofilm formation involves the attachment and accumulation of microbial cells, within a self-produced extracellular matrix, on a solid surface [2]. The inherently defensive character of the biofilm is demonstrated by enhanced persistence of bacteria grown in the sessile mode model *versus* bacteria grown planktonically, which makes most biofilm-associated infections difficult to eradicate, thus contributing to disease chronicity [3], [4]. *Pseudomonas aeruginosa*, a ubiquitous bacterium in the nature, is an opportunistic pathogen that adheres to surfaces and forms protective biofilms [3]. In addition, multidrug-resistant *P. aeruginosa* is a leading cause of nosocomial infection worldwide, ranking first among all nosocomial pathogens related to pneumonia in intensive care units in Brazil [5] and in the United States [6].

The challenge and difficulty in finding novel antibacterial agents with innovative mechanisms of action, including anti-adherent compounds, drive the search for antimicrobials toward vegetable sources. This is an appropriate choice because plants play an important role in the biosynthesis of natural products, providing chemical defense against environmental microbes through secondary metabolism, and because they can be considered as a therapeutic alternative in primary health care (ethnopharmacological knowledge). The Caatinga, a xeric shrub-dominated biome of northeastern Brazil, supports a high diversity of plant resources used as folk medicine. This region is known as an area of low economic development, which reflects poor access of the population to pharmaceutical drugs and, consequently, determines the treatment of illnesses based on the use of medicinal plants. The limited scientific basis for the biological properties of these plants prompted an interest in investigating species widespread in the Caatinga in more detail. Our ongoing efforts to evaluate their biological potential have revealed antibacterial and antibiofilm activities against the Gram-positive bacterium *Staphylococcus epidermidis* for several plant species [7] and against *P. aeruginosa* for a reduced number of plants (unpublished data).

In this context, tannins have attracted attention due to their biological and physiological properties [8]. The tannins are complex mixtures of oligomers widely distributed in plants and foods which are yielded by polymerization of flavan-3-ol units (condensed tannins) or by esterification of several gallic acid residues to a carbohydrate core, mainly glucose (hydrolysable tannins) [9], [10]. The polymerization degree and oxygenation pattern of tannins are related with their biological properties [9], [11], making relevant their structural identification. In several cases, the isolation of tannins is very intricate, therefore mixtures are being successfully analyzed by mass spectrometry (MS), such as Matrix-Assisted Laser Desorption/Ionization (MALDI) [12], [13]. The couplings between different flavan-3-ol units have been proposed with the MS data and there are few studies based in MS/MS data to identify tannins, even though the structural identification seems to be more reliable using the fragmentation pathway [14], [15].

This study aimed to investigate the activity of stem-bark extracts of *Anadenanthera colubrina*, *Commiphora leptophloeos* and *Myracrodruon urundeuva* and their ability to control the growth and prevent biofilm formation of *P. aeruginosa* using bioguided fractionation. The bioactive compounds were purified and further analyzed by MALDI-MS/MS in order to identify the structures.

## Materials and Methods

### Plant material

Stem barks were collected at a national park, Parque Nacional do Catimbau (PARNA do Catimbau), located in the state of Pernambuco, northeastern Brazil, between July and August 2009, under authorization of the responsible authority *Instituto Chico Mendes de Conservação da Biodiversidade* (ICMBio) using the license SISBIO 16.806. The taxonomic identification was confirmed at the herbarium of *Instituto Agronômico de Pernambuco* (IPA), where the vouchers were deposited (Table 1). As reported by official authorities in Brazil the species used in this study are not endangered or protected in the state of Pernambuco, where the sampling occurred. The extracts were prepared as previously described [7].

**Table 1:** Stem-bark plant species from the Brazilian Caatinga: MIC, CFU/mL and biofilm formation assessed by crystal violet assay (mean ± SD) for *Pseudomonas aeruginosa*.

Family	Scientific name	Popular name	Voucher	MIC (mg/mL)	CFU/mL (log)	Biofilm formation at 6 h (%)	Biofilm formation at 24 h (%)	Biofilm formation at 48 h (%)
Fabaceae -Mimosoideae	*Anadenantheracolubrina* (Vell.) Brenan *var. colubrina*	Angico	IPA 84039	2.5	8.7±0.06	13.7±4.4*	35.7±3.2*	108.4±19.1
Burseraceae	*Commiphora leptophloeos* (Mart.) J.B.Gillett	Imburana, amburana, imburana de cambão	IPA 84037	1.0	8.5±0.02	47.4±5.1*	70.6±1.6*	147.0±14.5*
Anacardiaceae	*Myracrodruon urundeuva* Allemão	Aroeira, aroeira-do-sertão	IPA 84059	4.0	8.8±0.06	20.7±2.0*	40.2±7.9*	176.7±15.5*

CFU: colony-forming units, MIC: minimum inhibitory concentration,, SD: standard deviation.

* Statistically different compared to untreated samples.

Experiments were carried out in triplicate. Untreated samples were considered as 100% of biofilm formation and presented log CFU/mL of 8.7±0.2.

### Bacterial strain and culture conditions

*Pseudomonas aeruginosa* ATCC 27853 was grown overnight on Mueller-Hinton (MH) agar (Oxoid Ltd., England, UK) at 37°C. A bacterial suspension of 3×10^8^ colony-forming units (CFU)/mL in 0.9% NaCl was used in the assays.

### Minimum inhibitory concentration (MIC) and bacterial viability

Bacterial growth was assessed as the difference between optical density at 600 nm (OD_600_) at the end (6 h) and at the beginning (0 h) of incubation time, in 96-well microtiter plates (Costar 3599, Corning, Inc., USA). In each well, 80 µL of the bacterial suspension, 80 µL of the aqueous extract (concentration ranging from 0.5 to 4.0 mg/mL in the wells) and 40 µL of tryptone soya broth (TSB) (Oxoid Ltd., England, UK) were added. MIC was defined as the lowest concentration of samples able to restrict bacterial growth to a level lower than 0.04 at OD_600_. Serial dilutions of the MIC wells were performed and spread on MH agar plates. After incubation (37°C, overnight), the CFU/mL was obtained, in order to determine the viability of bacterial cells. As references for bacterial growth and viability, the extracts were replaced with water (negative control) or with gentamicin sulfate (Sigma-Aldrich Co., USA) (positive control).

### Antibacterial activity kinetics

A kinetic study was performed to assess the effect of extracts (at concentrations of 1/4xMIC, 1/2xMIC, MIC, and 2xMIC) upon *P. aeruginosa* according to the incubation time, as previously described in Section 2.3. OD_600_ was measured at 0, 3, 6, 9, 24, 30, 48, 52, and 72 h after incubation (37°C). Samples were replaced with sterile water as a control for bacterial growth. To avoid the interference of sample color in all results obtained by OD evaluations, the samples were incubated in TSB and sterile saline (without inoculum) and the arithmetic means of OD readings were corrected for each extract (by subtracting OD without inoculum from OD with inoculum for each incubation time). Erythromycin (Sigma-Aldrich Co., USA) was used as control for bacteriostatic action. The results are expressed as mean ± standard deviation (SD) of 4 wells for each extract concentration and for each incubation time.

### Biofilm formation assay

Biofilm formation was evaluated using the crystal violet assay in 96-well microtiter plates [7]. The incubation period at 37°C was 6, 24, and 48 h. To represent 100% of biofilm formation (untreated sample), the extracts were replaced with sterile water. Values higher than 100% represented stimulation of biofilm formation in comparison with the untreated sample. Since does not exist a commercially available non-biocidal compound possessing antibiofilm activity, we can not apply a positive control to antibiofilm activity.

### Microscopic analysis

#### Scanning electron microscopy (SEM)

*Pseudomonas aeruginosa* biofilms were grown in 96-well microtiter plates (37°C during 6, 24, and 48 h) with a piece of Permanox™ slide (Nalge Nunc International, USA), as described in Section 2.3. The samples were prepared and examined according to Trentin et al [7].

#### Transmission electron microscopy (TEM)

Tubes containing 800 µL of the bacterial suspension, 800 µL of the aqueous extract (MIC concentration) and 400 µL of TSB were incubated (37°C, 6 h). In untreated samples, sterile water was added instead of samples. The bacteria were harvested by centrifugation, fixed in glutaraldehyde and paraformaldehyde solution, and, subsequently, in 2% osmium tetroxide. The pellet was dehydrated in an ascending series of acetone concentrations and cells were embedded in acetone:EmBed™ resin, homogenized by rotation and polymerized. Ultrathin sections were contrasted with uranyl acetate and lead citrate and imaged with a JEOL JEM-1200 EX II electron microscope (JEOL Ltd, Tokyo, Japan).

#### Fluorescence microscopy

Samples were prepared as described in Section 2.6.2. After incubation, they were stained with LIVE/DEAD BacLight Bacterial Viability Kit (Life Technologies, USA). In this assay, the SYTO-9 and propidium iodide (PI) stains compete for binding to the bacterial nucleic acid. SYTO-9 labels cells with both damaged and intact membranes (green cells), whereas PI penetrates only in the cells with damaged membranes (red cells), reducing the fluorescence of SYTO-9. The samples were observed in the AxioVert 200 fluorescence microscope using the AxioVision AC software (Carl Zeiss MicroImaging Inc, Germany), and image overlays were obtained using ImageJ software.

### Hemolytic assay

This assay was performed as previously described [16], using human venous blood. All the blood donors were healthy researchers and students who signed specific form for consent to participate in the study. The Universidade Federal do Rio Grande do Sul Ethical Committee approved all documents, procedures and project under authorization number 19346. All forms are stored in specific place in the laboratory. The extracts were tested at MIC and, as reference samples, we used water (for baseline values) and *Quillaja saponaria* saponins (Sigma-Aldrich Co., USA) at 0.25 mg/mL (for 100% hemolysis). To avoid the interference of sample color, a blank sample of extracts and phosphate-buffered saline (PBS) (without erythrocytes) was developed. The assay was calculated as follows: 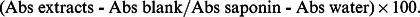


### Purification of proanthocyanidins

The crude aqueous extracts (100 mg) were dissolved in water (500 µL) and subjected to column chromatography (10×150 mm) packed with Sephadex™ LH-20 (Sigma-Aldrich Co., USA) successively until to obtain the appropriate amount of fractionated sample. Water was used as the first eluent, followed by 30% methanol, 50% methanol, 100% methanol, 10% acetone, 30% acetone, 50% acetone, 70% acetone, and 100% acetone, resulting in fractions coded as F1–F9. Additional data are available as supporting information (Table S1).

### MALDI-MS and MALDI-MS/MS analyses

High-resolution mass spectrometry (MS) analyses were performed using an UltrafleXtreme MALDI-TOF/TOF equipment (Bruker Daltonics, Bremen, Germany). A mixture of peptides was used for external and internal calibration (peptide calibration standard II [Bruker Daltonics]: bradykinin 1–7, angiotensin II and I, substance P, bombesin, renin substrate, ACTH clip 1–17, ACTH clip 18–39, and somatostatin 28). The ions were generated by irradiation with a nitrogen laser (337 nm) and accelerated at 20 kV. For MS analyses, the experimental conditions were: pulsed ion extraction of 100 ns, laser frequency of 1000 Hz, reflectron mode, positive ion mode, and 600 laser shots were averaged to record a mass spectrum. In addition, the selected ions were accelerated to 19 kV in the LIFT cell for MS/MS analyses. The matrix of choice was DHB (2,5-dihydroxybenzoic acid) at 20 mg/mL (in 30% acetonitrile [ACN] and 70% H_2_O with 0.1% trifluoroacetic acid). All samples were suspended in ACN∶H_2_O (3∶7) and mixed with DHB containing 0.1 M solution of NaCl. These mixtures (1 µL) were spotted onto a ground stainless steel MALDI target. The compounds were identified by MS data, fragmentation pathway and accurate mass measurements using the internal calibrant (standard peptide mixture).

### Statistical analysis

Biological assays were carried out in triplicate. Data differences in relation to the untreated samples were analyzed by the Student *t* test, and p≤0.05 was considered to be significant.

## Results

### MIC and viability determinations for stem-bark extracts

The MIC of all three stem-bark aqueous extracts against *P. aeruginosa* was determined (Table 1). CFU counting was used to determine the viability of 6 h-treated cells at MIC. Statistical analysis indicated that previously treated and untreated bacterial suspensions were equivalent regarding CFU/mL, suggesting bacteriostatic activity, i.e., when bacterial growth is inhibited (at MIC), the cells present viability (Table 1).

### Kinetic analysis of antibacterial activity

**Figure 1:**
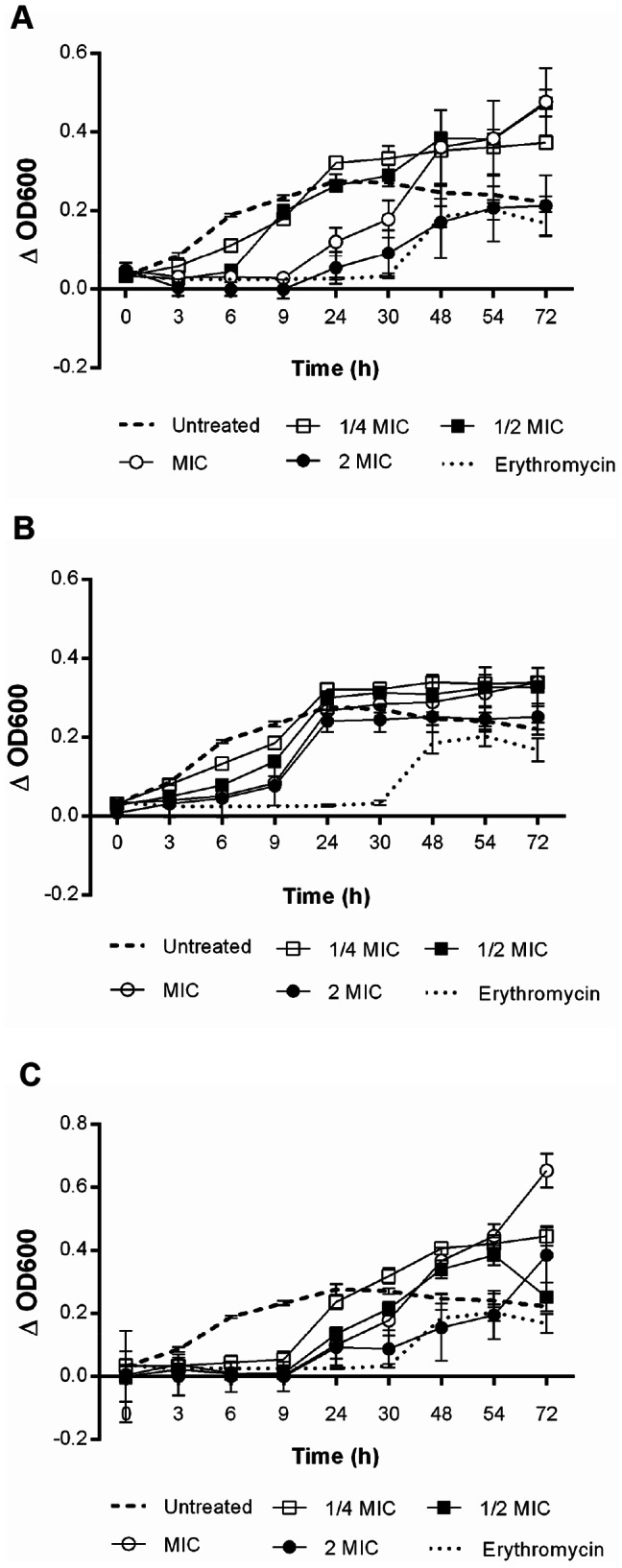
Kinetics of antibacterial activity obtained from *P.* *aeruginosa* exposed to stem-bark extracts, at four concentrations. (A) *A. colubrina*, (B) *C. leptophloeos*, and (C) *M. urundeuva*.

To observe the effect of extracts upon *P. aeruginosa* growth according to incubation time, a kinetic study was performed (Fig. 1). In this set of experiments, there was a significant decrease in bacterial growth within a short period of exposure to all extract concentrations (except for 1/4xMIC of *A. colubrina* and *C. leptophloeos* – Fig. 1A–B). Bacterial growth remained inhibited or low up to 9 h after incubation. After 24 h, extract-treated bacteria started to grow, achieving values similar to those obtained with untreated cells. Reinforcing our former result, a similar dynamic profile was observed with erythromycin, a bacteriostatic agent against *P. aeruginosa*.

### Activity upon biofilm formation

**Figure 2:**
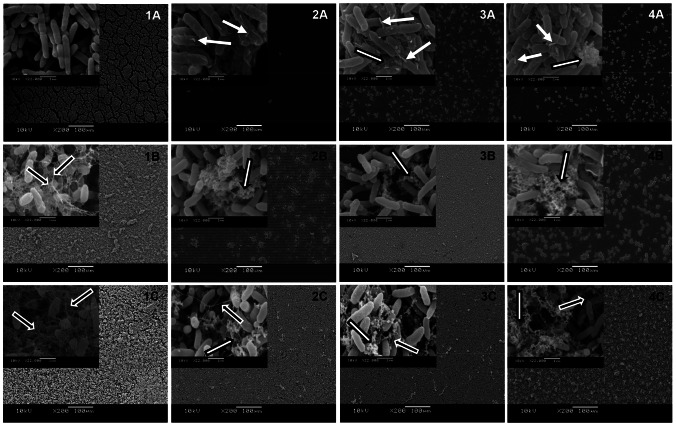
Scanning electron microscopy (SEM) images of biofilms upon Permanox™. Untreated *P. aeruginosa* biofilms (1). Extract-treated biofilms of *A. colubrina* – 2.5 mg/mL (2), *C. leptophloeos* – 1.0 mg/mL (3), and *M. urundeuva* – 4.0 mg/mL (4). Incubation time of 6 h (A), 24 h (B), and 48 h (C). Scale bars: 200× magnification in the images (insert of 22000× magnification). White arrows: morphological changes as stalked nubs; black arrows with white outline: matrix production; white arrows with black outline: extract material.

The effect of extracts at MIC upon *P. aeruginosa* biofilm formation on the polystyrene surface, using crystal violet assay, is shown in Table 1. Biofilm formation was strongly prevented in 6 h-treated bacteria in relation to untreated cells. After 24 h of treatment, the extracts remained able to inhibit biofilm formation, although with a loss in their absolute antibiofilm activity by 20% when compared to inhibition at an earlier stage (6 h). According to this assay, no antibiofilm effect was observed at 48 h of incubation. Unlikely, *C. leptophloeos* and *M. urundeuva* extracts stimulated biofilm formation (Table 1). These results were corroborated by morphological features observed using different microscopic techniques. SEM images showed rod-shaped cells of untreated *P. aeruginosa* grown on the Permanox surface, forming a dense and uniform biofilm (untreated biofilms) (Fig. 2 images 1A–C), covered with an extracellular matrix (Fig. 2 images 1B and 1C). In contrast, 6 h-treated biofilms displayed lesser adherent bacteria and only slight aggregation, reducing bacterial agglomerates to small clusters (Fig. 2 images 2A, 3A, and 4A). At 24 h of incubation, we could still observe a low number of bacterial clusters deficient in matrix production compared to the untreated sample (Fig. 2 images 2B and 4B), while for *C. leptophloeos*-treated cells a larger amount of aggregated cells was observed, but without the presence of a matrix (Fig. 2 image 3B). After 48 h of treatment, matrix production was extremely poor; the bacterial cluster architecture varied among samples and was different from the typical biofilm shape of the untreated *P. aeruginosa*. However, it was possible to observe that cell agglomerates had completely covered the surface (Fig. 2 images 2C, 3C, and 4C).

### Transmission electron microscopy (TEM) and fluorescence microscopy (FM)

**Figure 3:**
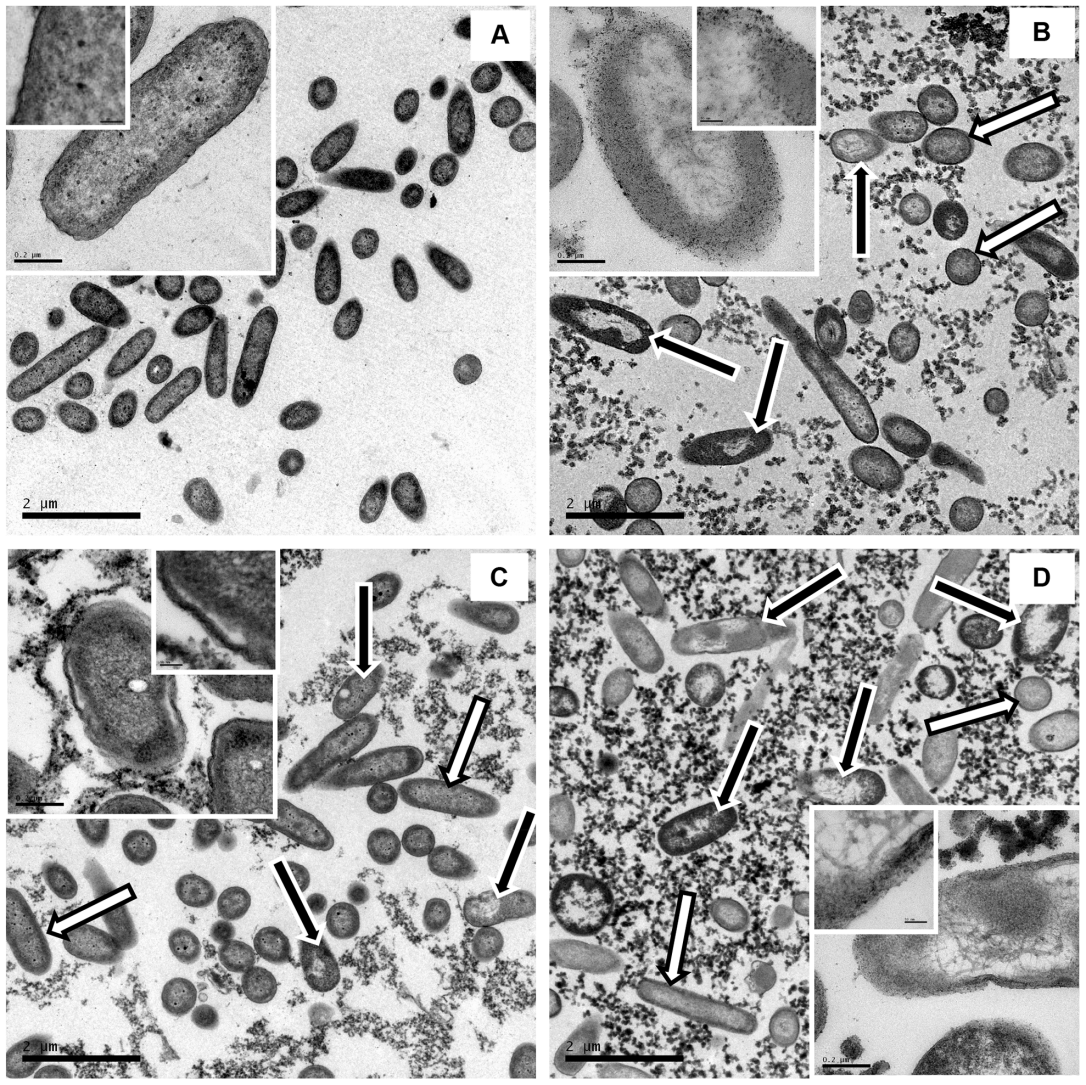
Transmission electron microscopy (TEM). (A) untreated and extract-treated *P. aeruginosa* cells, at minimum inhibitory concentration (MIC), of (B) *A. colubrina*, (C) *C. leptophloeos*, and (D) *M. urundeuva*. Scale bars: 15000× magnification in the images (inserts: 120000× magnification and 500000× magnification). Black arrows with white outline: vacuoles in the cells; white arrows with black outline: normal morphology.

**Figure 4:**
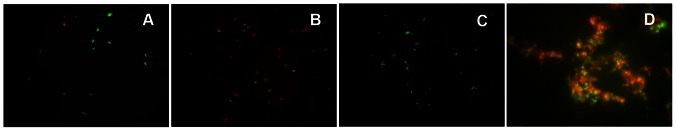
Fluorescence microscopy (FM) images. (A) untreated and extract-treated *P. aeruginosa* cells, at minimum inhibitory concentration (MIC), of (B) *A. colubrina*, (C) *C. leptophloeos*, and (D) *M. urundeuva* (1000× magnification).

On TEM micrographs, untreated cells exhibited an undisturbed cytosol and intact cell envelope (cytoplasmic membrane and cell wall) (Fig. 3A). In contrast, extracts at MIC induced ultrastructural modifications in *P. aeruginosa* cells (Fig. 3B–D). At 15000× magnification, images revealed that all three extracts were able to promote intense vacuolization in several cells (as signaled by black arrows with white outline), although some cells with normal morphology remained present (white arrows with black outline). Regarding *A. colubrina* and *M. urundeuva*, cell deformation and disrupted cell wall could also be observed (Fig. 3B and D, in the inserts). In *C. leptophloeos*-treated cells, in addition to an injured cell wall, we could observe vacuoles dispersed throughout the cytoplasm and within the periplasm of cells (Fig. 3C, in the inserts). FM images reinforced these results: at MIC, both viable (green) and nonviable (red) cells could be observed, indicating a damaged cytoplasmic membrane in several treated cells (Fig. 4B–D).

### Hemolytic activity

We carried out a simple model to assess injury in human cells and preliminary toxicity of extracts. At MIC, *A. colubrina* and *M. urundeuva* caused 4.3±1.9% and 13.1±0.5% of hemolysis, respectively while *C. leptophloeos* was not hemolytic (0±1.2%). The microscopic analysis showed erythrocyte integrity and absence of erythrocyte aggregation (data not shown).

### Bioguided fractionation

**Figure 5:**
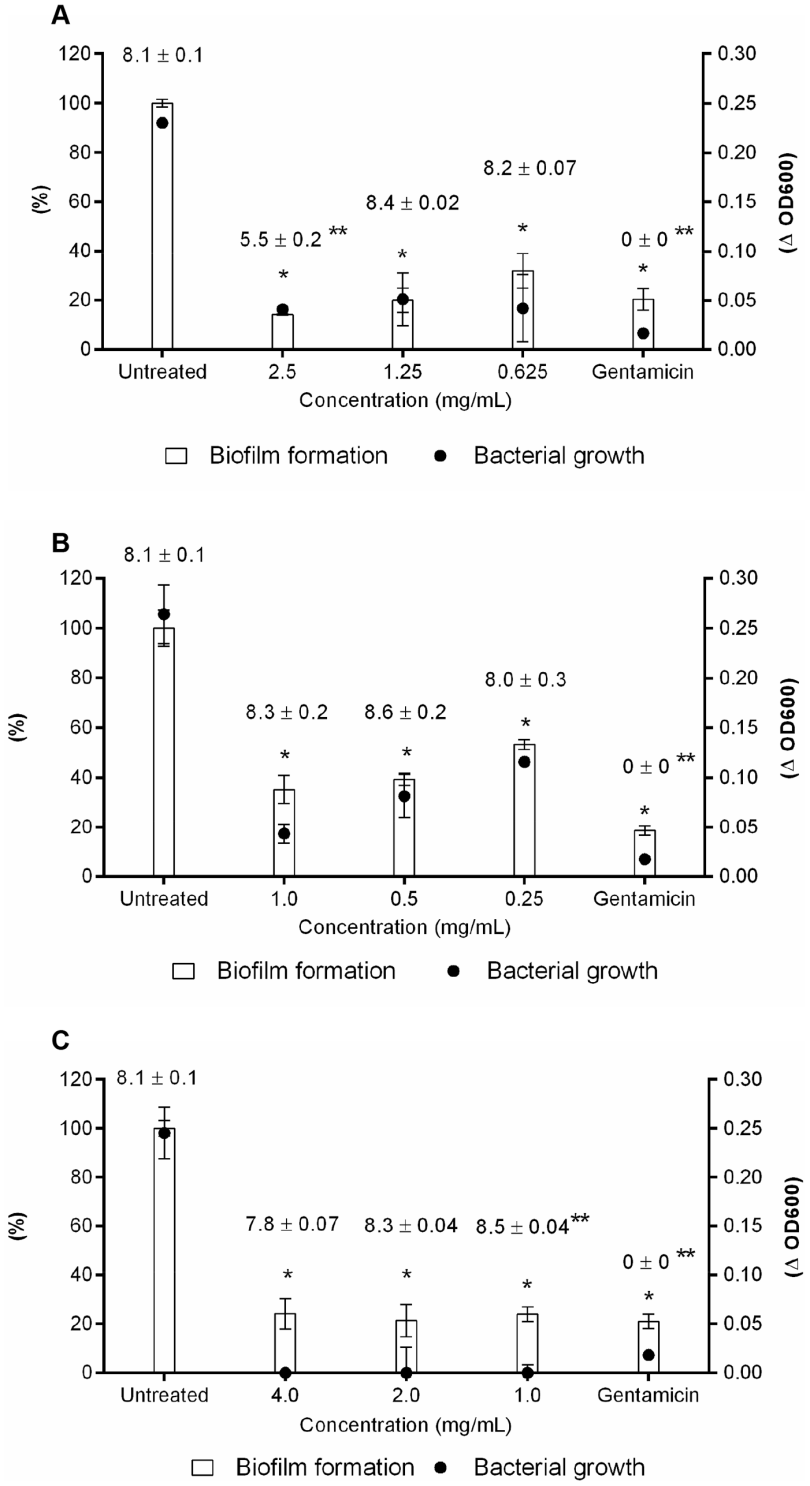
Effect of tannins (F7) on *P.* *aeruginosa* biofilm formation, bacterial growth, and viability. (A) *A. colubrina*, (B) *C. leptophloeos*, and (C) *M. urundeuva*. Gentamicin was used as a positive control. Numbers on top of bars are the mean values ± standard deviation (SD) of log CFU/mL. *Represents statistical difference in growth and biofilm formation compared to the untreated sample and **represents statistical difference in CFU/mL compared to the untreated sample.

The aqueous extracts were fractionated on Sephadex LH-20. The eluted fractions (F1–F9) were tested for *P. aeruginosa* antibiofilm and antibacterial activities at three concentrations: MIC, 1/2xMIC and 1/4xMIC of the crude extracts, at 6 h of incubation. For all plants, the F7 fraction provided the same activity observed for the crude extracts (Fig. 5). The fraction obtained from *A. colubrina* allowed a biofilm formation of 14, 20, and 32% at 2.5, 1.25, and 0.625 mg/mL, respectively, with growth inhibition in all tested concentrations (Fig. 5A). The same profile was observed for F7 obtained from *M. urundeuva* at all concentrations tested, in which biofilm formation was limited to 23%, accompanied by absence of growth (Fig. 5C). Regarding F7 from *C. leptophloeos*, at 1.0 mg/mL, bacterial growth was suppressed, with biofilm formation of about 35%. In *C. leptophloeos*, when fraction concentration decreased to 0.25 mg/mL, *P. aeruginosa* began to grow and biofilm inhibition decreased (54% of biofilm formation) (Fig. 5B). The number of CFU/mL after F7 treatments was determined and bacteriostatic effects were observed in all concentrations tested for the three plants evaluated (Fig. 5A–C).

### MALDI study of tannin fractions

**Figure 6:**
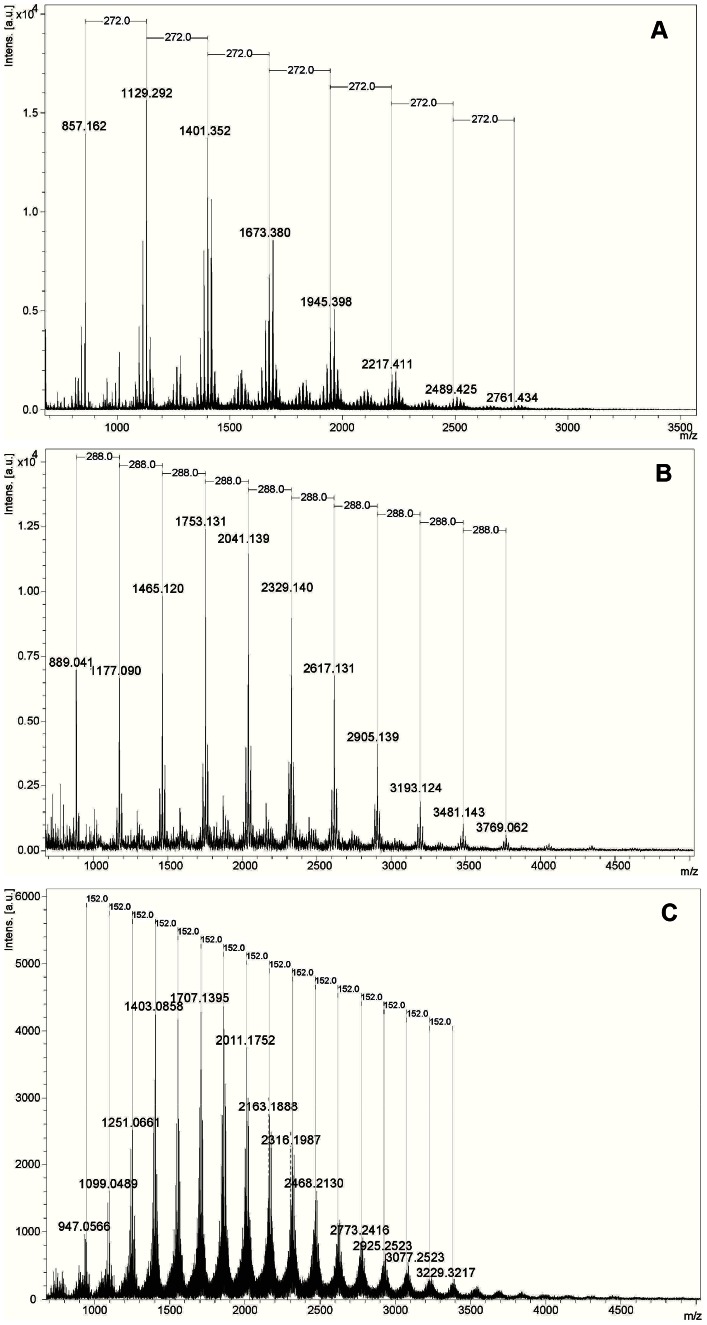
Mass spectra (positive ion mode) of F7 fractions. (A) *A. colubrina*, (B) *C. leptophloeos*, and (C) *M. urundeuva*. In the images, the majoritarian series of each fraction is highlighted (*A. colubrina* – series B and *C. leptophloeos* – series A).

**Figure 7:**
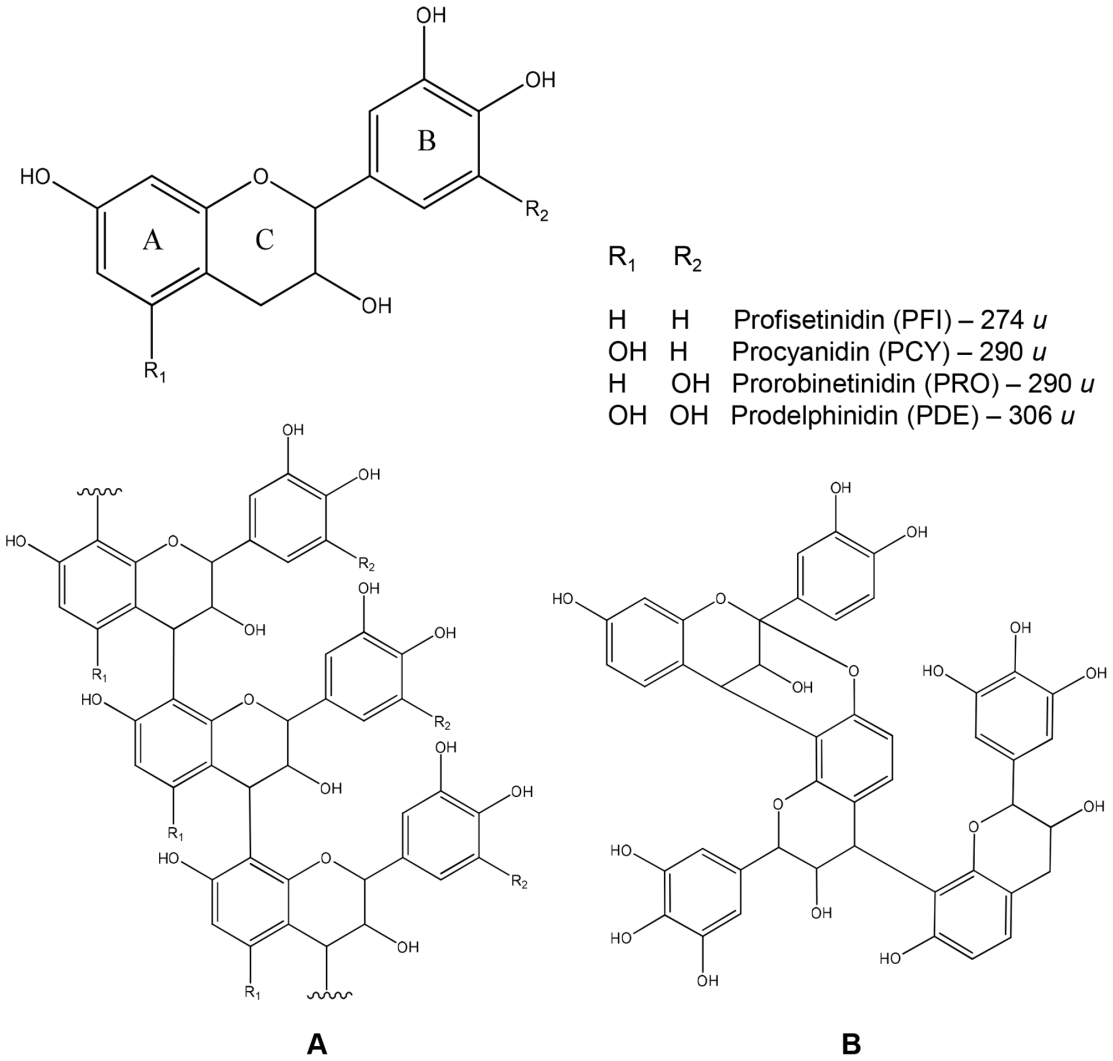
Polymeric structure of the condensed tannin purified from the plants studied. Typical linear condensed tannin with a B-type linkage (A) and an A-type linkage (*m/z* 871 [M+Na]^+^) identified from *C. leptophloeos* (B). Profisetinidin (PFI), procyanidin (PCY), prorobinetinidin (PRO), and prodelphinidin (PDE).

Figure 6 shows the MALDI-TOF spectra of the polymeric tannin mixture in the F7 fractions from *A. colubrina*, *C. leptophloeos*, and *M. urundeuva*. The molecular formulae of tannins were obtained from measured accurate masses (Fig. S3 and Fig. S15), and the MS/MS data were important to establish and confirm the units of flavan-3-ol or galloyl bound in oligomers. All ions and fragment ions were cationized by sodium. Proanthocyanidins (or condensed tannins) were identified from *A. colubrina* fraction yielding units mainly of profisetinidin type (Fig. 7) up to 10 units (Table 2 – series B), and from *C. leptophloeos* fraction, in which the units are mainly of prorobinetinidin type (Fig. 7) reaching up to 13 units (Table 2 – series A–C). Conversely, *M. urundeuva* fraction is composed of hydrolyzable tannins which are gallic acid derivatives, being esterified to a glucose residue.

**Table 2:** Distribution of polyflavonoid oligomers by MALDI-TOF for tannin F7 fractions of *Anadenanthera colubrina* and *Commiphora leptophloeos*.

Fraction F7		[M+Na]^+^ (error)	MF	Compound
	Series A	841.2139 (4.3 ppm)	C_45_H_38_O_15_	3 PFI
		1113.2831 (3.9 ppm)	C_60_H_50_O_20_	4 PFI
		1385.3545 (5.2 ppm)	C_75_H_62_O_25_	5 PFI
		1657.4131 (1.6 ppm)	C_90_H_74_O_30_	6 PFI
		1929.4889 (2.4 ppm)	C_105_H_86_O_35_	7 PFI
		2201.5526 (6.2 ppm)	C_120_H_98_O_40_	8 PFI
		2473.6316 (4.2 ppm)	C_135_H_110_O_45_	9 PFI
	Series B	857.2089 (4.3 ppm)	C_45_H_38_O_16_	PFI – PCY - PFI
		1129.2660 (6.0 ppm)	C_60_H_50_O_21_	PFI - PCY - 2 PFI
		1401.3444 (1.6 ppm)	C_75_H_62_O_26_	PFI - PCY - 3 PFI
		1673.4018 (5.3 ppm)	C_90_H_74_O_31_	PFI - PCY - 4 PFI
		1945.4764 (1.4 ppm)	C_105_H_86_O_36_	PFI - PCY - 5 PFI
		2217.5378 (4.4 ppm)	C_120_H_98_O_41_	PFI - PCY - 6 PFI
		2489.6035 (5.0 ppm)	C_135_H_110_O_46_	PFI - PCY - 7 PFI
		2761.6966 (4.4 ppm)	C_150_H_122_O_51_	PFI - PCY - 8 PFI
	Series C	1417.3433 (4.4 ppm)	C_75_H_62_O_27_	PFI - PCY - 2 PFI - PCY
		1689.3964 (5.4 ppm)	C_90_H_74_O_32_	PFI - PCY - 2 PFI - PCY - PFI
		1961.4630 (5.6 ppm)	C_105_H_86_O_37_	PFI - PCY - 2 PFI - PCY - 2 PFI
		2233.5266 (7.1 ppm)	C_120_H_98_O_42_	PFI - PCY - 2 PFI - PCY - 3 PFI
		2505.6230 (4.8 ppm)	C_135_H_110_O_47_	PFI - PCY - 2 PFI - PCY - 4 PFI
	Series D	873.2014 (1.5 ppm)	C_45_H_38_O_17_	PFI - PCY - PRO
		1145.2718 (2.8 ppm)	C_60_H_50_O_22_	PFI - PCY - PRO - PFI
	Series A	889.1903 (5.3 ppm)	C_45_H_38_O_18_	3 PRO
		1177.2547 (3.2 ppm)	C_60_H_50_O_24_	4 PRO
		1465.3119 (6.8 ppm)	C_75_H_62_O_30_	5 PRO
		1753.3925 (4.2 ppm)	C_90_H_74_O_36_	6 PRO
		2041.4558 (3.5 ppm)	C_105_H_86_O_42_	7 PRO
		2329.5124 (0.2 ppm)	C_120_H_98_O_48_	8 PRO
		2617.5827 (2.8 ppm)	C_135_H_110_O_54_	9 PRO
		2905.6428 (1.4 ppm)	C_150_H_122_O_60_	10 PRO
		3193.7053 (0.1 ppm)	C_165_H_134_O_66_	11 PRO
		3481.7596 (1.7 ppm)	C_180_H_146_O_72_	12 PRO
		3769.8211 (2.1 ppm)	C_195_H_158_O_78_	13 PRO
	Series B	871.1876 (3.6 ppm)	C_45_H_36_O_17_	PFI - PRO - PRO*
		1159.2381 (8.4 ppm)	C_60_H_48_O_23_	PFI - PRO - 2 PRO*
		1447.3182 (4.8 ppm)	C_75_H_60_O_29_	PFI - PRO - 3 PRO*
		1735.3695 (3.0 ppm)	C_90_H_72_O_35_	PFI - PRO - 4 PRO*
		2023.4327 (2.6 ppm)	C_105_H_84_O_41_	PFI - PRO - 5 PRO*
		2311.5065 (2.2 ppm)	C_120_H_96_O_47_	PFI - PRO - 6 PRO*
		2599.5567 (3.1 ppm)	C_135_H_108_O_53_	PFI - PRO - 7 PRO*
		2887.6290 (0.3 ppm)	C_150_H_120_O_59_	PFI - PRO - 8 PRO*
		3175.7055 (4.4 ppm)	C_165_H_132_O_65_	PFI - PRO - 9 PRO*
		3463.7^NO^	C_180_H_144_O_71_	PFI - PRO - 10 PRO*
		3751.8^NO^	C_195_H_156_O_77_	PFI - PRO - 11 PRO*
	Series C	1193.2623 (7.5 ppm)	C_60_H_50_O_25_	3 PRO - PDE
		1481.3236 (4.6 ppm)	C_75_H_62_O_31_	3 PRO - PDE - PRO
		1769.3789 (0.7 ppm)	C_90_H_74_O_37_	3 PRO - PDE - 2 PRO
		2057.4309 (6.1 ppm)	C_105_H_86_O_43_	3 PRO - PDE - 3 PRO
		2345.4921 (6.3 ppm)	C_120_H_98_O_49_	3 PRO - PDE - 4 PRO
		2633.5869 (6.3 ppm)	C_135_H_110_O_55_	3 PRO - PDE - 5 PRO
		2921. 6171(5.3 ppm)	C_150_H_122_O_61_	3 PRO - PDE - 6 PRO
		3209.6808 (5.1 ppm)	C_165_H_134_O_67_	3 PRO - PDE - 7 PRO
		3497.8^NO^	C_180_H_146_O_73_	3 PRO - PDE - 8 PRO
		3785.8^NO^	C_195_H_158_O_79_	3 PRO - PDE - 9 PRO

MF: molecular formula, PFI: profisetinidin, PCY: procyanidin, PRO: prorobinetinidin, PDE: prodelphinidin.

* A-type, NO: not observed with internal calibrant (low intensity).

The mass spectra of *A. colubrina* bioactive fraction (F7) showed four polymeric series (A, B, C and D, Table 2), mostly composed of units of profisetinidin showed through consecutive losses 272 *u* (Table 2, Fig. 6A and Fig. S1). The main differences among the series occurred in lowest-mass oligomers where other units are linked. All the polymeric series of condensed tannins were proposed by MS/MS and peaks correspond to the loss one, two, three and more units were observed. In the majoritarian polymeric series B (*m/z* 857, 1129, 1401, 1673, 1945, 2217, 2489, 2761) (Fig. 6A and Table 2 – series B), the MS/MS of *m/z* 857 yielded the fragment ions *m/z* 585 and 295 (Fig. S5), the latter confirmed profisetinidin as the starter unit and the former was related to procyanidin addition. The fragment ions *m/z* 705 and 433 (Fig. S5) represented the loss of 152 *u* due to Retro Diels-Alder (RDA) fission (C-ring fragmentation pathway) and confirmed the B-ring substituents of the proposed units (procyanidin and profisetinidin). This loss was also observed for the other ions of series B (Fig. S10, Fig. S11 and Fig. S13). Regarding the minority series (A, C and D), is possible to observe that series A is comprised of oligomers formed only by profisetinidin units (Table 2 and Fig. S1, Fig. S4, Fig. S7 and Fig. S9). Differently of series B, the series C presents, additionally, one procyanidin residue (288 *u*) in the structure of its oligomers (Table 2 and Fig. S12 and Fig. S14). The series D is composed of up to four units in the following sequence: profisetinidin, procyanidin, prorobinetinidin and profisetinidin (Table 2, Fig. S6 and Fig. S8), where the loss of 168 *u* due to RDA fission from ion at *m/z* 873 confirmed the B-ring substituents of prorobinetinidin unit (Fig. S6). The polymeric series of *A. colubrina* showed differences in 16 *u*, such as *m/z* 841, 857 and 873, which represents oxygen (hydroxyl) due the mass difference between 288 *u* monomers (procyanidin and/or prorobinetinidin) and 272 *u* monomers (prodelfinidin); additionally, all compounds presented B type linkage (Fig. 7A).

In the bioactive fraction (F7) from *C. leptophloeos* three polymeric series (A, B and C) were found, being rich in prorobinetinidin units as demonstrated by consecutive losses of 288 *u* (Table 2, Fig. 6B and Fig. S2). The main polymeric series was yielded with exclusively prorobinetinidin units (Fig. 6B and Table 2 – series A) exhibiting B-type linkage pattern (Fig. 7A) and their compounds were determined through MS/MS data (Fig. S17, Fig. S19, Fig. S22, Fig. S23). The minority series B differs from the others since A-type linkage is found. The starter unit in this series is a profisetinidin linked to repeat units of prorobinetinidin. The peak *m/z* 1159 yielded the fragments *m/z* 1007, 989, 871, 719, 581, 429, and 311 in the MS/MS spectrum (Fig. S18), similarly to what was observed for the fragmentation pathway of the ion *m/z* 871 (Fig. S16) and for the other peaks from this series (Fig. S21). The compound with *m/z* 871 was produced by coupling one profisetinidin and two prorobinetidins, the second unit with an A-type linkage (Fig. 7B). Thus, the compounds of polymeric series B present a difference of 2 *u* compared with similar tannin showing B-type linkage. Series C was formed by repeat units of prorobinetinidin and only one prodelphinidin, which was confirmed by MS/MS data, where the loss of 168 *u* due to RDA fission confirmed the prodelphinidin unit (Fig. S20).

MS/MS analysis of the bioactive fractions from *A. colubrina* (Fig. S4–S14) and from *C. leptophloeos* (Fig. S16–S23) showed the same loss patterns for structural characterization of condensed tannins, such as the losses due to RDA fission and flavan-3-ol units; therefore only the classical fragmentations are addressed in this work.

The bioactive fraction (F7) from *M. urundeuva* was also analyzed by MALDI-TOF, and hydrolyzable tannins were observed in its composition. The MS spectrum showed peaks with increments of 152 *u* (Fig. 6C) and MS/MS spectra showed consecutive losses of gallic acid units attached to glucose () confirming the presence of gallotannins in this fraction, as observed in a recent study [17]. However, the structural elucidation of gallotannin series was not possible, since the MS/MS data were inconclusive.

## Discussion

The ability to impair bacterial adhesion represents an ideal strategy to combat bacterial pathogenesis, given its importance in the early stages of the infectious process. In addition, bacterial adhesion blockade is suitable as a prophylactic intervention to prevent infection [18]. Therefore, the use of natural agents that can successfully inhibit cell attachment is a promising tool for reduction of bacterial colonization on several surfaces [19]. In recent years, studies have reported anti-adhesive or antibiofilm activities of compounds, which are related to their antimicrobial properties [20]–[24].

Plants produce many compounds that are biologically active, either as part of their normal program of growth and development or in response to pathogen attack or stress. Traditionally, *A. colubrina*, *C. leptophloeos* and *M. urundeuva* have been used by communities in the Caatinga to treat infectious diseases, such as cough, bronchitis, influenza, urinary/liver diseases, ulcerative external lesions, and ovarian inflammation [25], [26].

In this study, we demonstrated that a highly complex mixture (Fig. 6, Fig. 7 and Table 2) of proanthocyanidins (mostly composed of profisetinidin in *A. colubrina* and prorobinetinidin in *C. leptophloeos*) and hydrolyzable tannins (consisting of gallic acid units in *M. urundeuva*) induced *P. aeruginosa* damage, providing bacteriostatic and anti-adhesive effects. Bacterial growth kinetic experiments revealed that inhibition of bacterial growth persisted up to 9 h post-incubation (Fig. 1). The bacteriostatic property was confirmed by cell counting (CFU/mL), after both aqueous extract and the tannin-enriched fraction F7 treatments (Table 1 and Fig. 5), and by recovering bacterial growth of extract- and erythromycin-treated cells, as indicated by OD_600_ measurements in the kinetic experiment (Fig. 1). At MIC, the extracts were also able to inhibit bacterial adhesion and prevent biofilm formation on the polystyrene surface for 6 and 24 h after incubation. However, this activity was lost after 48 h of incubation (Table 1). SEM was employed to improve understanding of the qualitative impact of the extracts upon the behavior of bacteria. We could observe that 6 h-treated cells presented morphological changes, such as stalked nubs (white arrows with black outline in Fig. 2), a phenotype that is also induced by amikacin and oxytetracycline [27]. This finding supports the hypothesis that morphological changes were due to the mechanisms of bacteria involved in protection against aggression from extracts. After 24 and 48 h of incubation (the latter characterizing the mature stage of biofilm development), untreated *P. aeruginosa* was enclosed by extracellular matrix while just the 48 h-treated cells were surrounded by a very discrete matrix (Fig. 2). Flemming and Wingender [28] demonstrated that matrix is essential for biofilm formation, which allows a lifestyle that is entirely different from the planktonic state, and concluded that there is no biofilm without a matrix. Based on these observations, we may suggest that *P. aeruginosa* agglomerates as visualized by SEM at 24 and 48 h of treatment could not be considered biofilm structures.

The level of bacterial membrane dysfunction could potentially result in cell death and may explain the rapid loss of viability observed in the kinetic experiments. Although TEM and FM showed no viable cells, these analyses also indicated the presence of cells without morphological alterations and with an intact membrane. These findings are consistent with Pankey and Sabath report [29], who highlighted that most agents characterized as bacteriostatic agents are able to kill some bacteria – often more than 90–99% of the inoculum, but this is not sufficient (>99.9%) to characterize them as bactericidal agents. Although *in vitro* bacteriostatic/bactericidal data may provide information on the potential action of antimicrobial agents, this is only one of many factors required to predict a favorable clinical outcome. Bacteriostatic agents have been effectively used in the treatment of endocarditis, meningitis, and osteomyelitis – indications that are often considered to require bactericidal activity [29]. Additionally, the bacterial membrane may be compromised during antimicrobial treatments, such as due to exposure to a bacteriostatic agent. The ultrastructural analysis of *P. aeruginosa* (Fig. 3) showed that extract-exposed cells presented excess vacuoles and a disrupted cell wall, when compared to untreated samples. FM results (Fig. 4) suggest that the extracts have anti-membrane activity, resulting in the disturbance of membrane structure in a large amount of cells, while displaying absence or very low toxicity against human erythrocytes, which corroborates the idea of a selective action of these tannins upon *P. aeruginosa* membrane.

The programmed death of some damaged cells may be beneficial to a multicellular bacterial community [30]. Thus, the occurrence of both viable and dying cells after extracts treatment could be understood as a suicide mission that would contribute to the maintenance of a population. There is strong evidence that genomic DNA released during *P. aeruginosa* lysis is a structural component of the biofilm matrix, supporting the idea that cell lysis contributes to the stability of the biofilm structure [31]. These findings are in agreement with our data, which show the development of *P. aeruginosa* clusters at 48 h of incubation, at the same time point when the potential of extracts to impair bacterial adhesion is decreased. The immune system is capable of eliminating pathogens that would otherwise persist in the presence of bacteriostatic agents, although the elimination of persister cells from biofilms by the immune system has not been specifically studied yet [30].

As a Gram-negative bacterium, *P. aeruginosa* has a cell wall consisting of a peptidoglycan layer and an additional outer membrane [32]. It should be noted that, in order to reach the cell membrane, tannins must cross the bacterial cell wall. Scalbert [33] has suggested that the cell wall probably fixes part of tannins, contributing to increase their MIC values. This observation is in agreement with our results, since MIC values for the extracts ranged from 1.0 to 4.0 mg/mL (Table 1) and for purified tannins (F7) the value was 1.0 mg/mL for *C. leptophloeos* and at least 0.625 mg/mL and 1.0 mg/mL for *A. colubrina* and *M. urundeuva*, respectively (Fig. 5).

This study represents an unprecedented report on phytochemical analysis, identifying tannins from three important plants used as folk medicine in Brazil: *A. colubrina*, *C. leptophloeos,* and *M. urundeuva*. It is also the first work to elucidate the tannin structure of plants from the genera *Anadenanthera* and *Commiphora*. Considering the origin of the plant material (stem-barks), the extraction method (aqueous maceration) and the data about the tannin content of these species [34], the achievement of tannins by bioguided fractionation was as expected. It is worth mentioning that, as previously reported by Almeida et al [35], Caatinga plants are exposed to high solar radiation in a semiarid environment, which favors the synthesis of phenolic derivatives, reinforcing the medicinal potential of plants from this biome.

In summary, we propose that tannins are able to inhibit biofilm formation by damaging the bacterial membrane and hindering matrix production, displaying bacteriostatic properties. Therefore, biofilm formation is prevented during minimal bacterial growth; when cells start to grow, they are able to attach to the surface and develop matrix-deficient cell clusters. The bacteriostatic activity against *P. aeruginosa* observed in this study provides a scientific basis which may justify some uses of these plants in traditional medicine. To the best of our knowledge, this is the first study to show the modulation of *P. aeruginosa* biofilm formation by the herein described bacteriostatic tannins and the first to report the identification of tannins from *A. colubrina* and *C. leptophloeos*. This fact highlights the importance of this widespread and abundant class of secondary metabolites in higher plants against one of the most challenging issues in the hospital setting: biofilm resilience.

## Supporting Information

Table S1 Yield (mg/%; w/w) of each fraction obtained from Sephadex LH-20 column in relation to the powdered plant material.
(TIF)

Figure S1 **Mass spectra (positive ion mode) of the fraction obtained from** ***A. colubrina*** **, presenting the series A, C and D, respectively.**
(TIF)

Figure S2 **Mass spectra (positive ion mode) of the fraction obtained from** ***C. leptophloeos*** **, presenting the series B and C, respectively.**
(TIF)

Figure S3 **Mass spectra (positive ion mode) of the fraction obtained from** ***A. colubrina*** **with internal calibrant (peptide calibration standard II).**
(TIF)

Figure S4 **MS/MS spectrum of ion** ***m/z*** **841 from** ***A. colubrina*** **.**
(TIF)

Figure S5 **MS/MS spectrum of ion** ***m/z*** **857 from** ***A. colubrina*** **.**
(TIF)

Figure S6 **MS/MS spectrum of ion** ***m/z*** **873 from** ***A. colubrina*** **.**
(TIF)

Figure S7 **MS/MS spectrum of ion** ***m/z*** **1113 from** ***A. colubrina*** **.**
(TIF)

Figure S8 **MS/MS spectrum of ion** ***m/z*** **1145 from** ***A. colubrina*** **.**
(TIF)

Figure S9 **MS/MS spectrum of ion** ***m/z*** **1385 from** ***A. colubrina*** **.**
(TIF)

Figure S10 **MS/MS spectrum of ion** ***m/z*** **1129 from** ***A. colubrina*** **.**
(TIF)

Figure S11 **MS/MS spectrum of ion** ***m/z*** **1401 from** ***A. colubrina*** **.**
(TIF)

Figure S12 **MS/MS spectrum of ion** ***m/z*** **1417 from** ***A. colubrina*** **.**
(TIF)

Figure S13 **MS/MS spectrum of ion** ***m/z*** **1673 from** ***A. colubrina*** **.**
(TIF)

Figure S14 **MS/MS spectrum of ion** ***m/z*** **1689 from** ***A. colubrina*** **.**
(TIF)

Figure S15 **Mass spectra (positive ion mode) of the fraction obtained from** ***C. leptophloeos*** **with internal calibrant (peptide calibration standard II).**
(TIF)

Figure S16 **MS/MS spectrum of ion** ***m/z*** **871 from** ***C. leptophloeos*** **.**
(TIF)

Figure S17 **MS/MS spectrum of ion** ***m/z*** **889 from** ***C. leptophloeos*** **.**
(TIF)

Figure S18 **MS/MS spectrum of ion** ***m/z*** **1159 from** ***C. leptophloeos*** **.**
(TIF)

Figure S19 **MS/MS spectrum of ion** ***m/z*** **1177 from** ***C. leptophloeos*** **.**
(TIF)

Figure S20 **MS/MS spectrum of ion** ***m/z*** **1193 from** ***C. leptophloeos*** **.**
(TIF)

Figure S21 **MS/MS spectrum of ion** ***m/z*** **1447 from** ***C. leptophloeos*** **.**
(TIF)

Figure S22 **MS/MS spectrum of ion** ***m/z*** **1465 from** ***C. leptophloeos*** **.**
(TIF)

Figure S23 **MS/MS spectrum of ion** ***m/z*** **2041 from** ***C. leptophloeos*** **.**
(TIF)

Figure S24 **MS/MS spectrum of ion** ***m/z*** **1403 from** ***M. urundeuva*** **.**
(TIF)
