# Supplementary figures and images for: Tannins Possessing Bacteriostatic Effect Impair Pseudomonas aeruginosa Adhesion and Biofilm Formation
Source: PLoS One. 2013 Jun 11;8(6):e66257. doi: 10.1371/journal.pone.0066257 (PMC3679062; doi:10.1371/journal.pone.0066257)

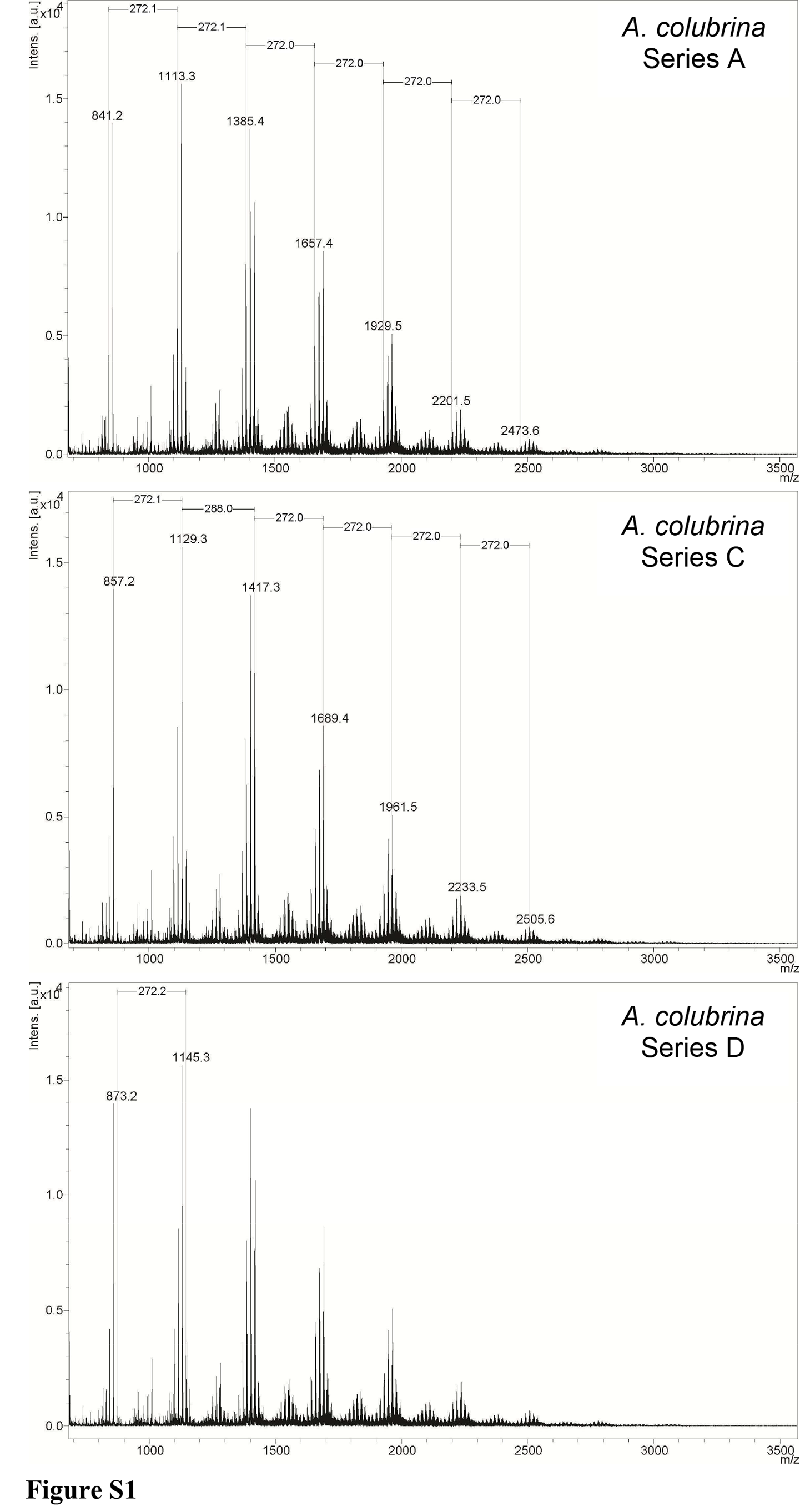

Supplement: Figure S1 — Mass spectra (positive ion mode) of the fraction obtained from A. colubrina , presenting the series A, C and D, respectively. (TIF) [file pone.0066257.s002.tif]

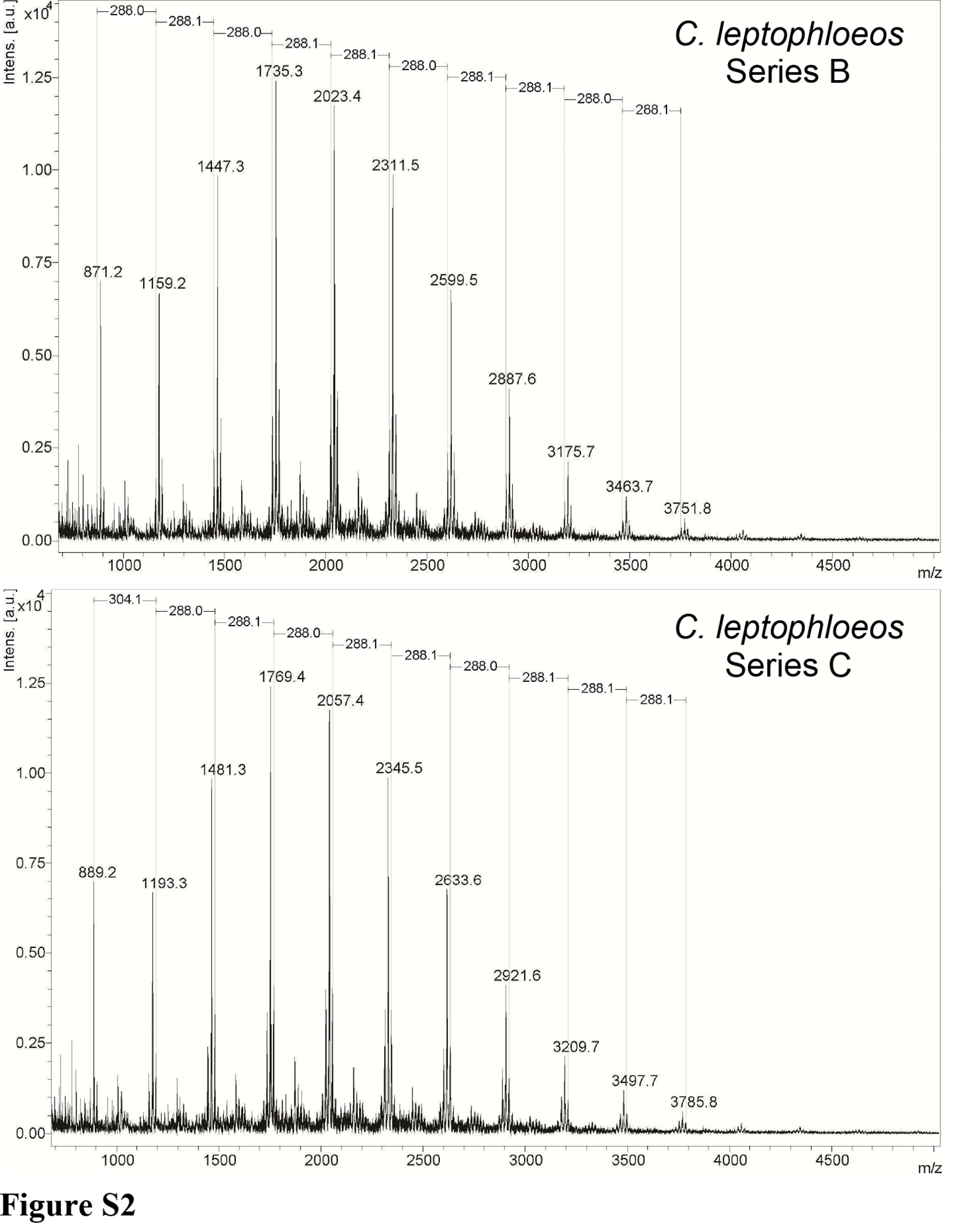

Supplement: Figure S2 — Mass spectra (positive ion mode) of the fraction obtained from C. leptophloeos , presenting the series B and C, respectively. (TIF) [file pone.0066257.s003.tif]

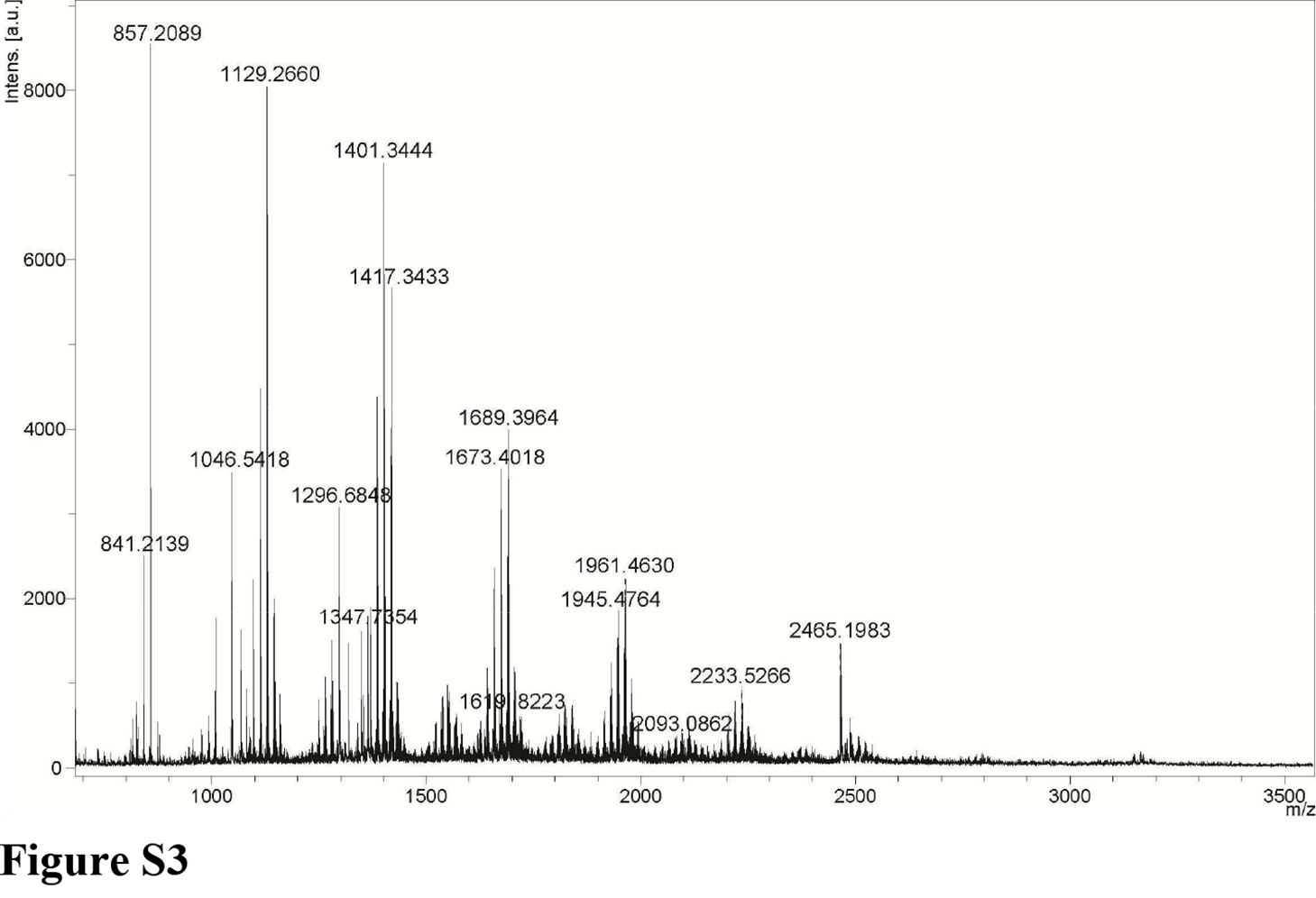

Supplement: Figure S3 — Mass spectra (positive ion mode) of the fraction obtained from A. colubrina with internal calibrant (peptide calibration standard II). (TIF) [file pone.0066257.s004.tif]

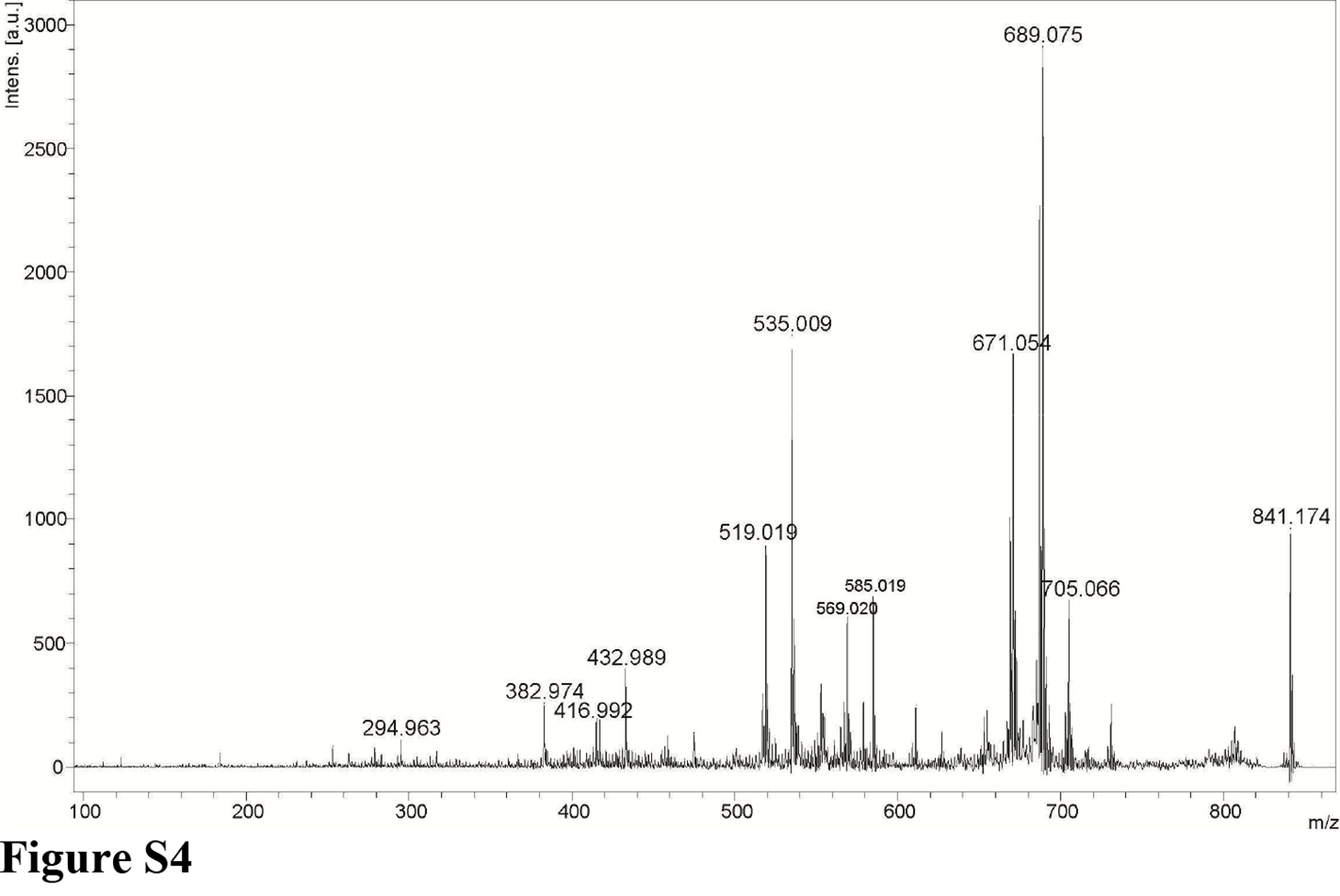

Supplement: Figure S4 — MS/MS spectrum of ion m/z 841 from A. colubrina . (TIF) [file pone.0066257.s005.tif]

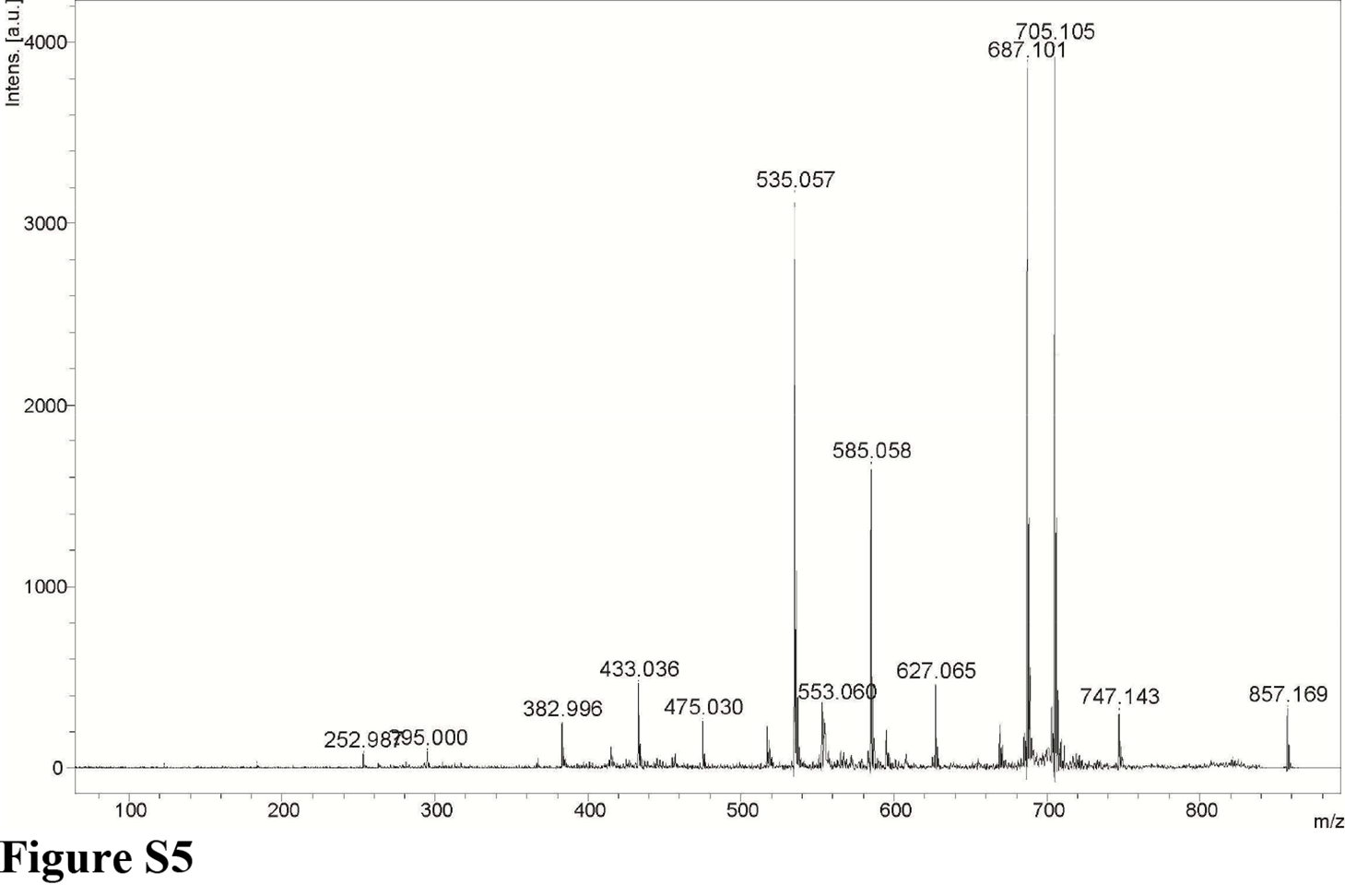

Supplement: Figure S5 — MS/MS spectrum of ion m/z 857 from A. colubrina . (TIF) [file pone.0066257.s006.tif]

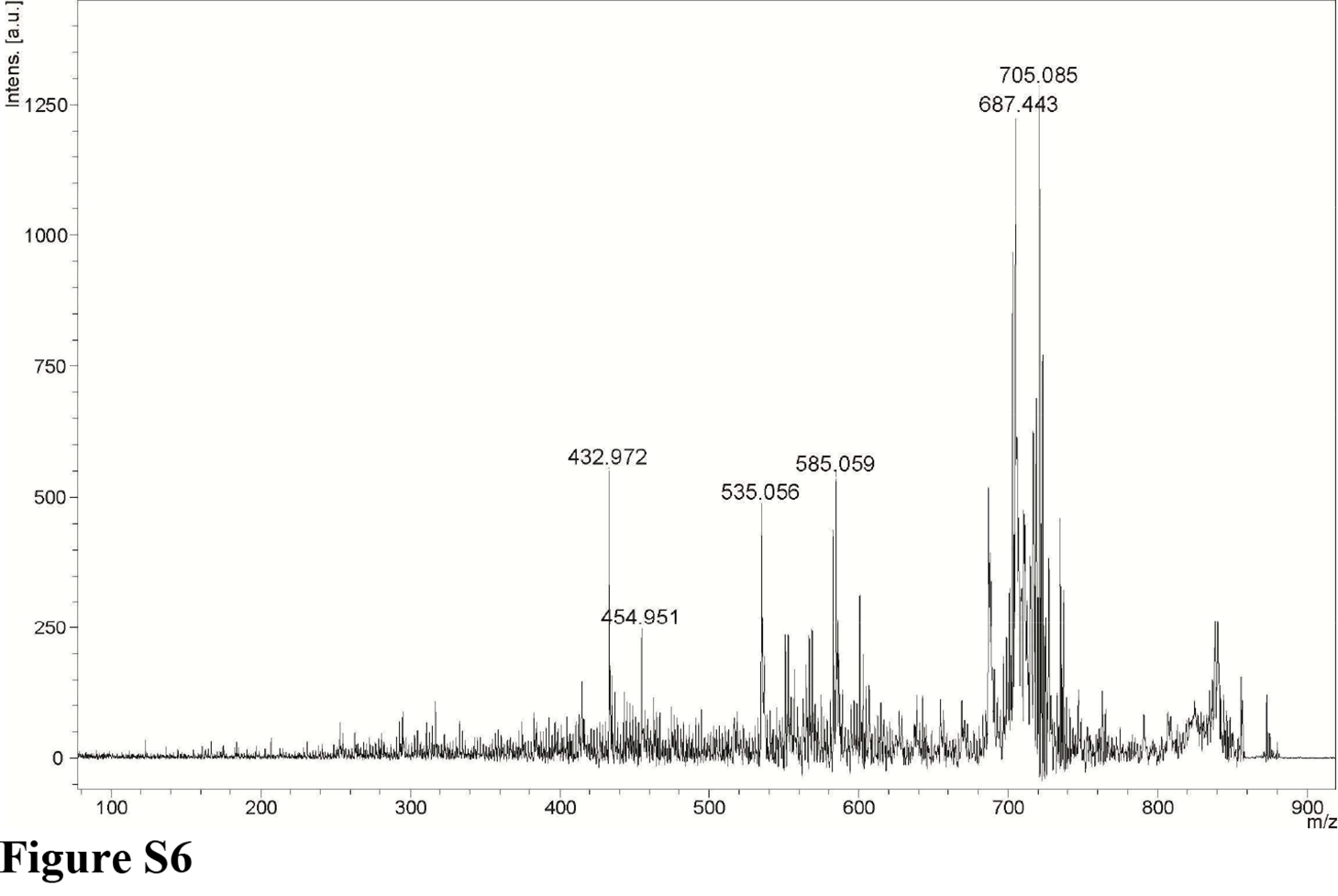

Supplement: Figure S6 — MS/MS spectrum of ion m/z 873 from A. colubrina . (TIF) [file pone.0066257.s007.tif]

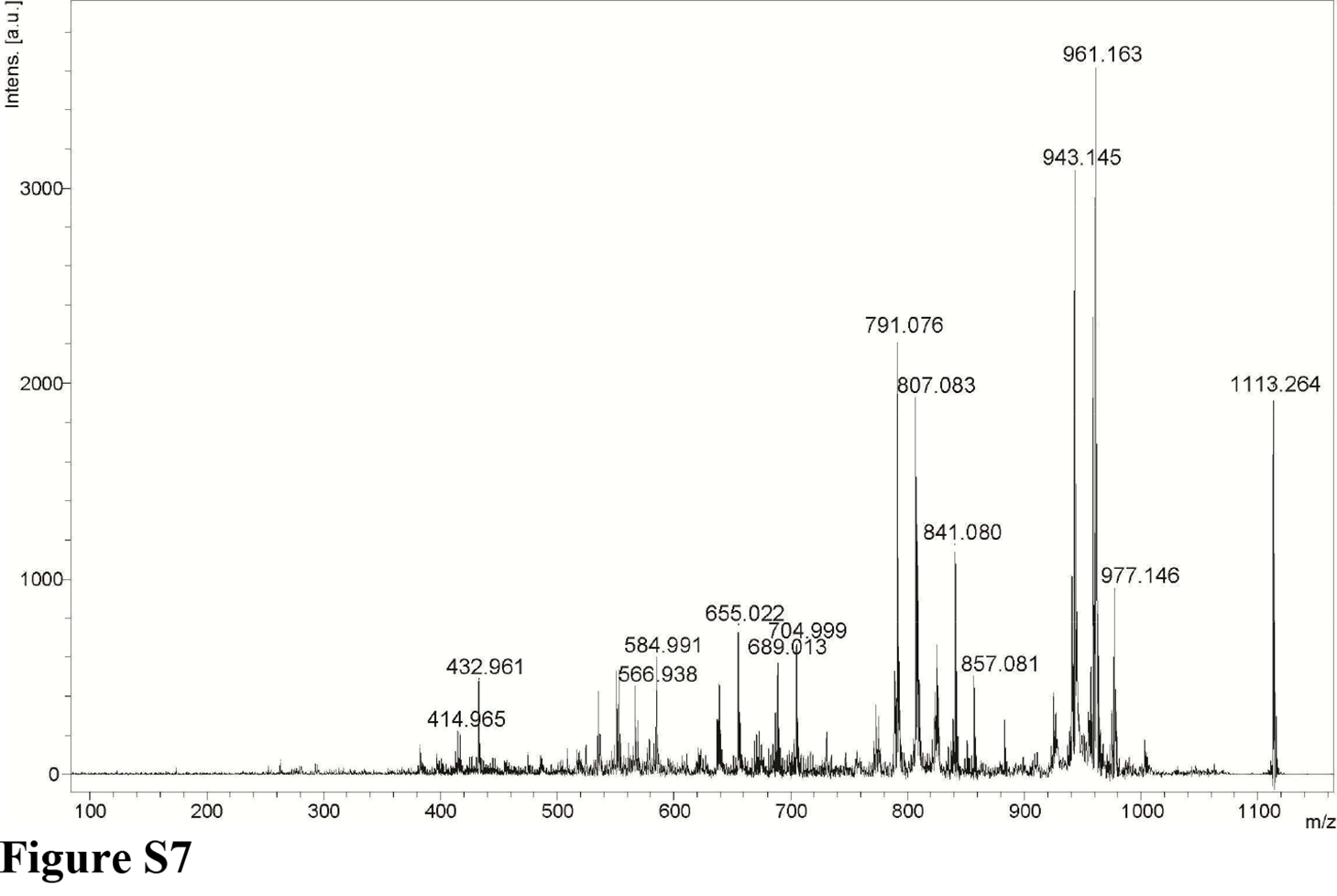

Supplement: Figure S7 — MS/MS spectrum of ion m/z 1113 from A. colubrina . (TIF) [file pone.0066257.s008.tif]

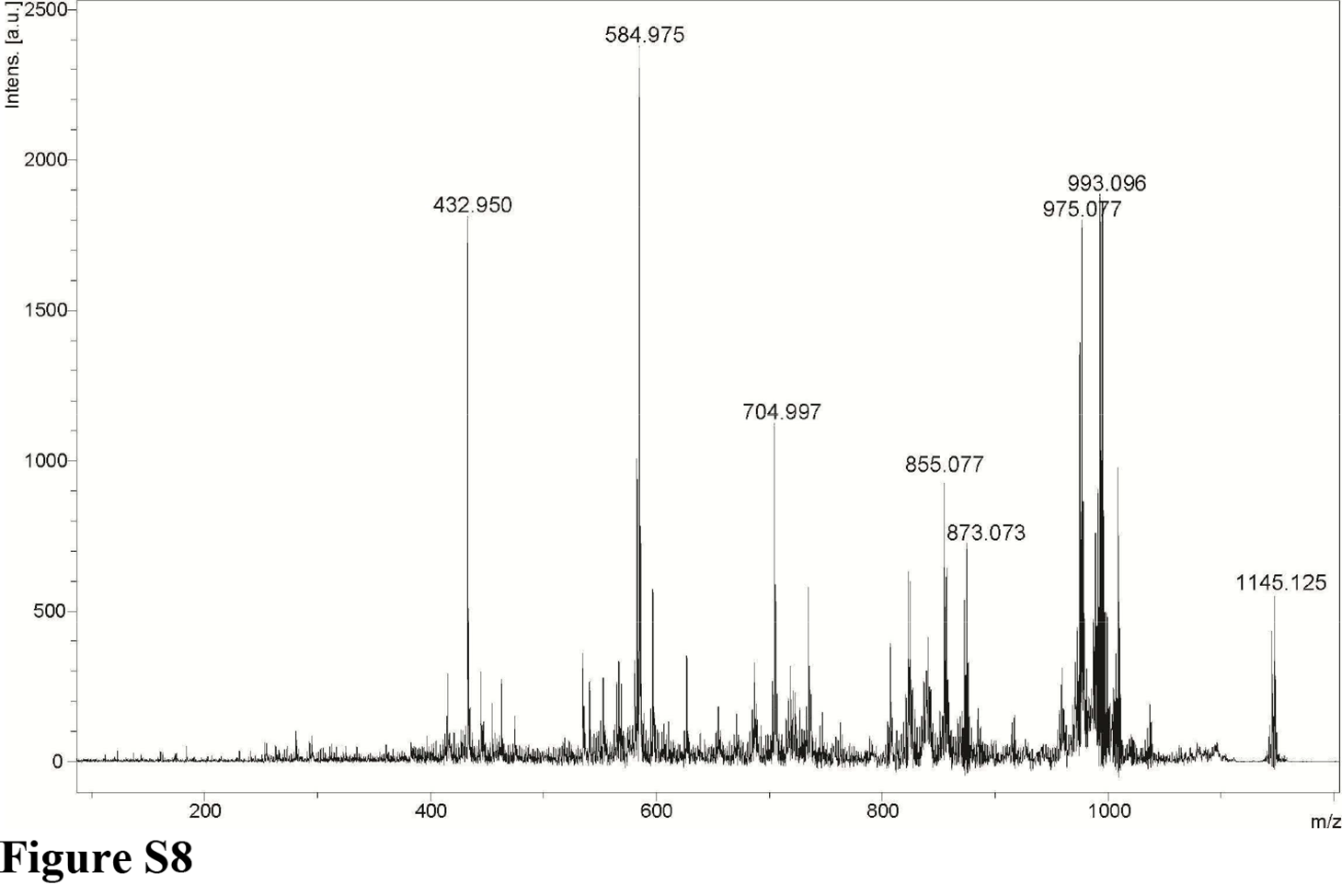

Supplement: Figure S8 — MS/MS spectrum of ion m/z 1145 from A. colubrina . (TIF) [file pone.0066257.s009.tif]

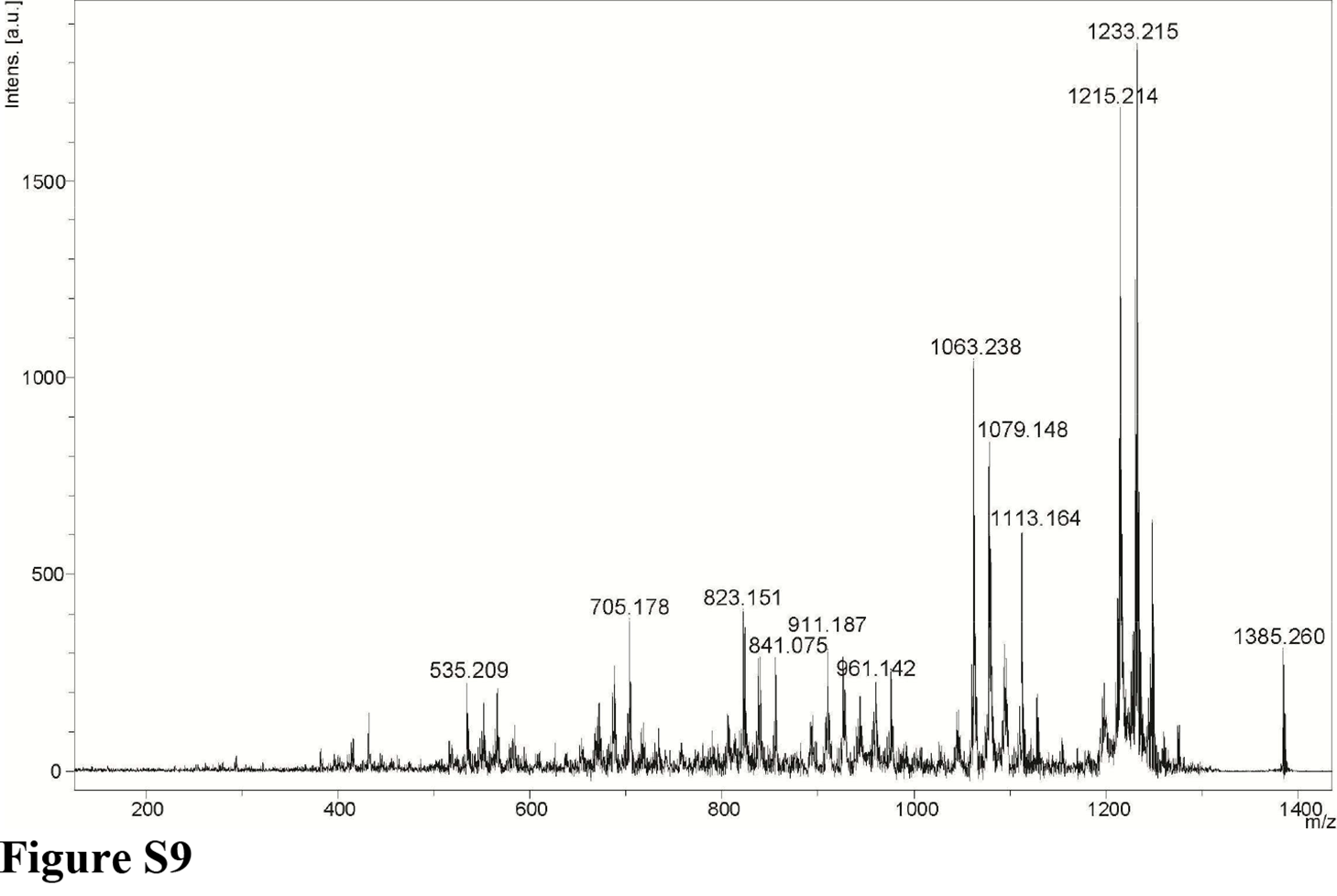

Supplement: Figure S9 — MS/MS spectrum of ion m/z 1385 from A. colubrina . (TIF) [file pone.0066257.s010.tif]

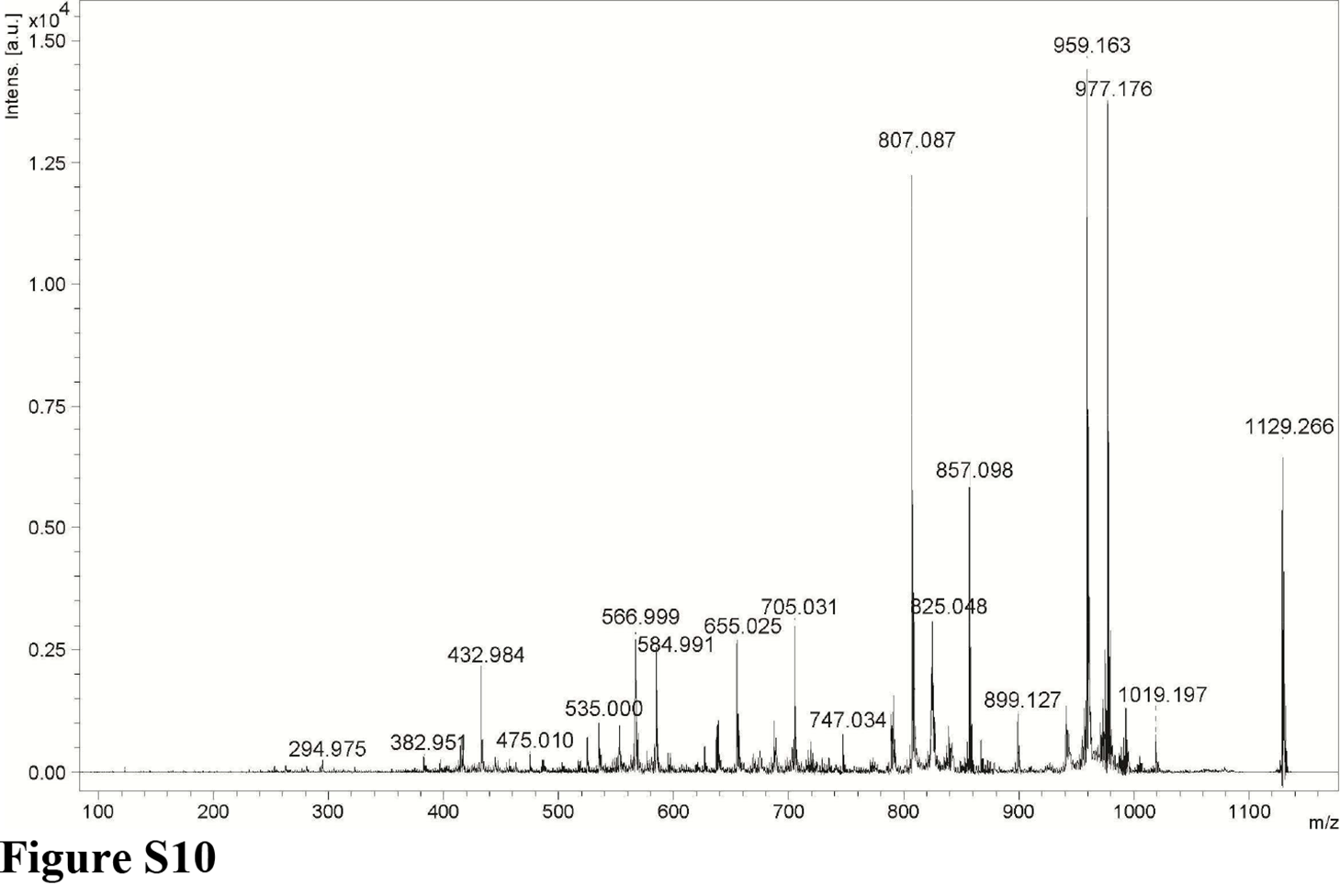

Supplement: Figure S10 — MS/MS spectrum of ion m/z 1129 from A. colubrina . (TIF) [file pone.0066257.s011.tif]

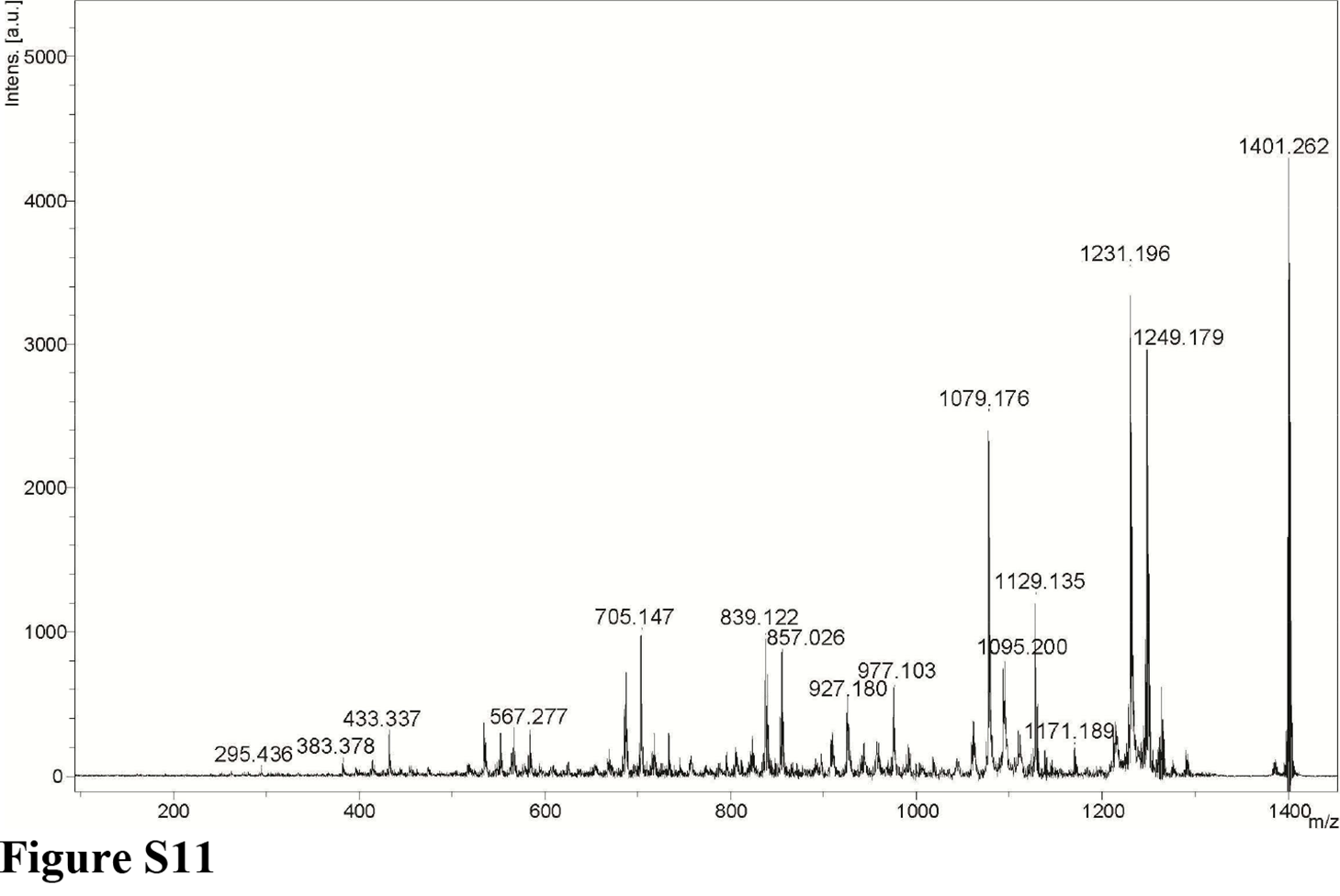

Supplement: Figure S11 — MS/MS spectrum of ion m/z 1401 from A. colubrina . (TIF) [file pone.0066257.s012.tif]

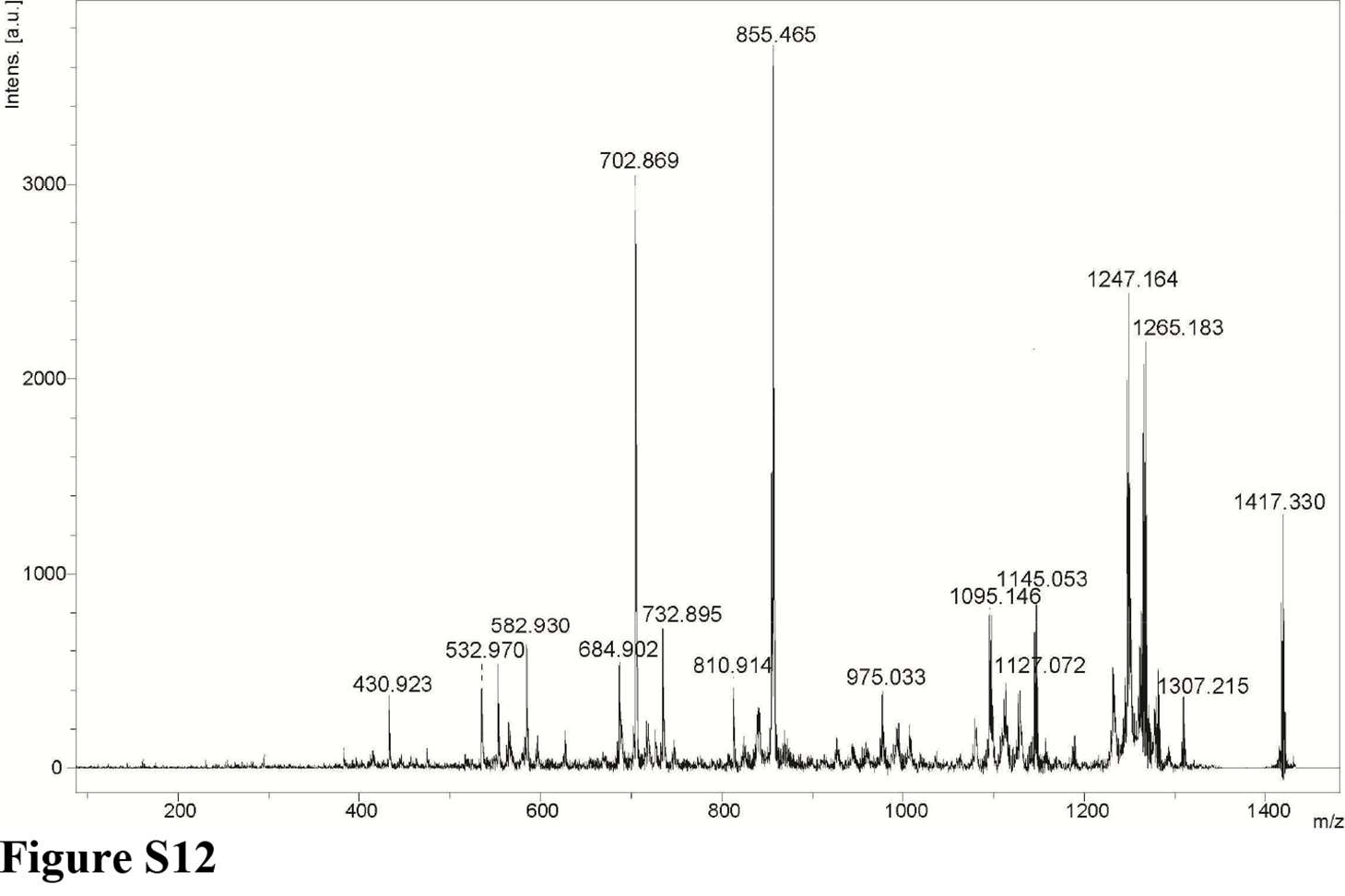

Supplement: Figure S12 — MS/MS spectrum of ion m/z 1417 from A. colubrina . (TIF) [file pone.0066257.s013.tif]

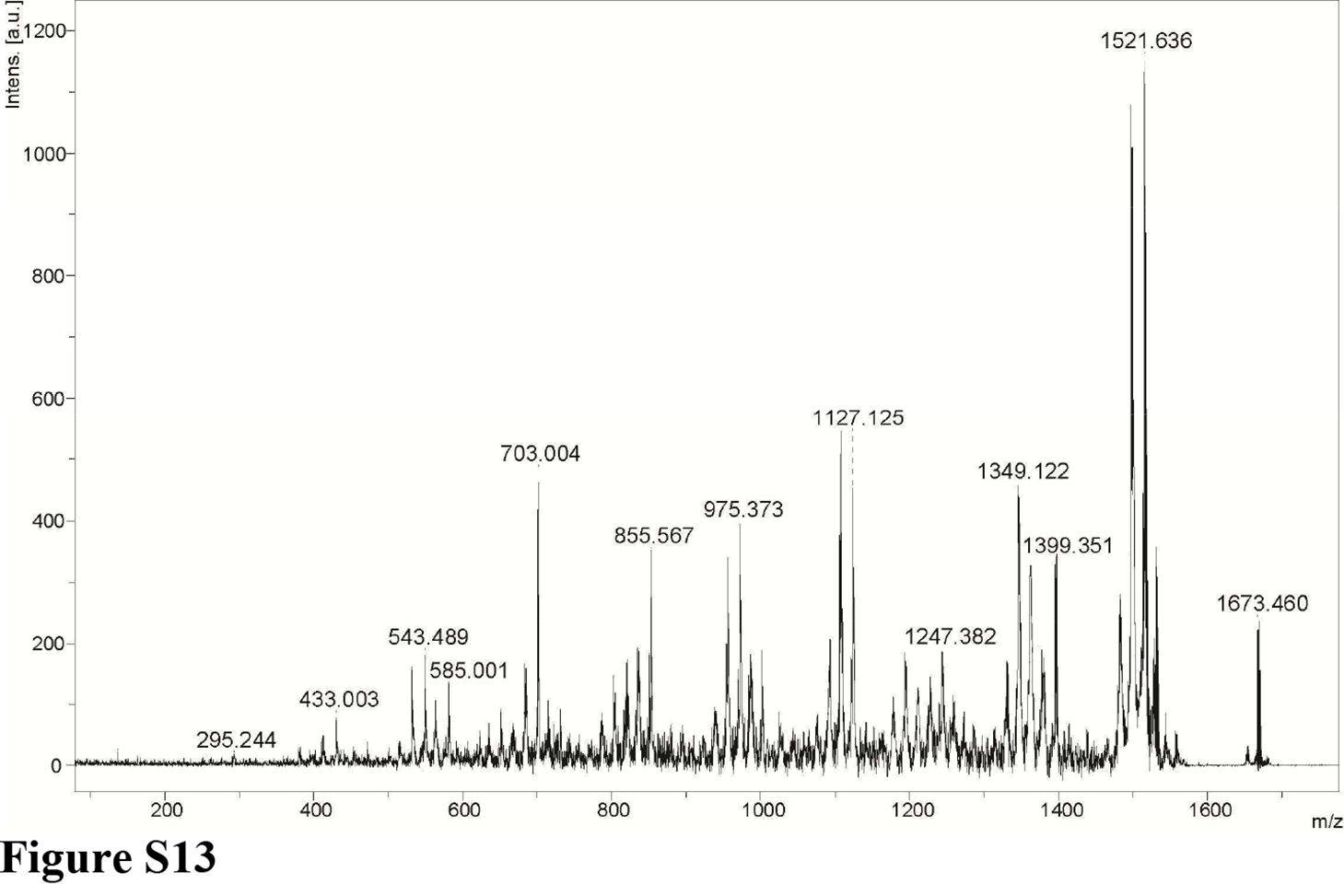

Supplement: Figure S13 — MS/MS spectrum of ion m/z 1673 from A. colubrina . (TIF) [file pone.0066257.s014.tif]

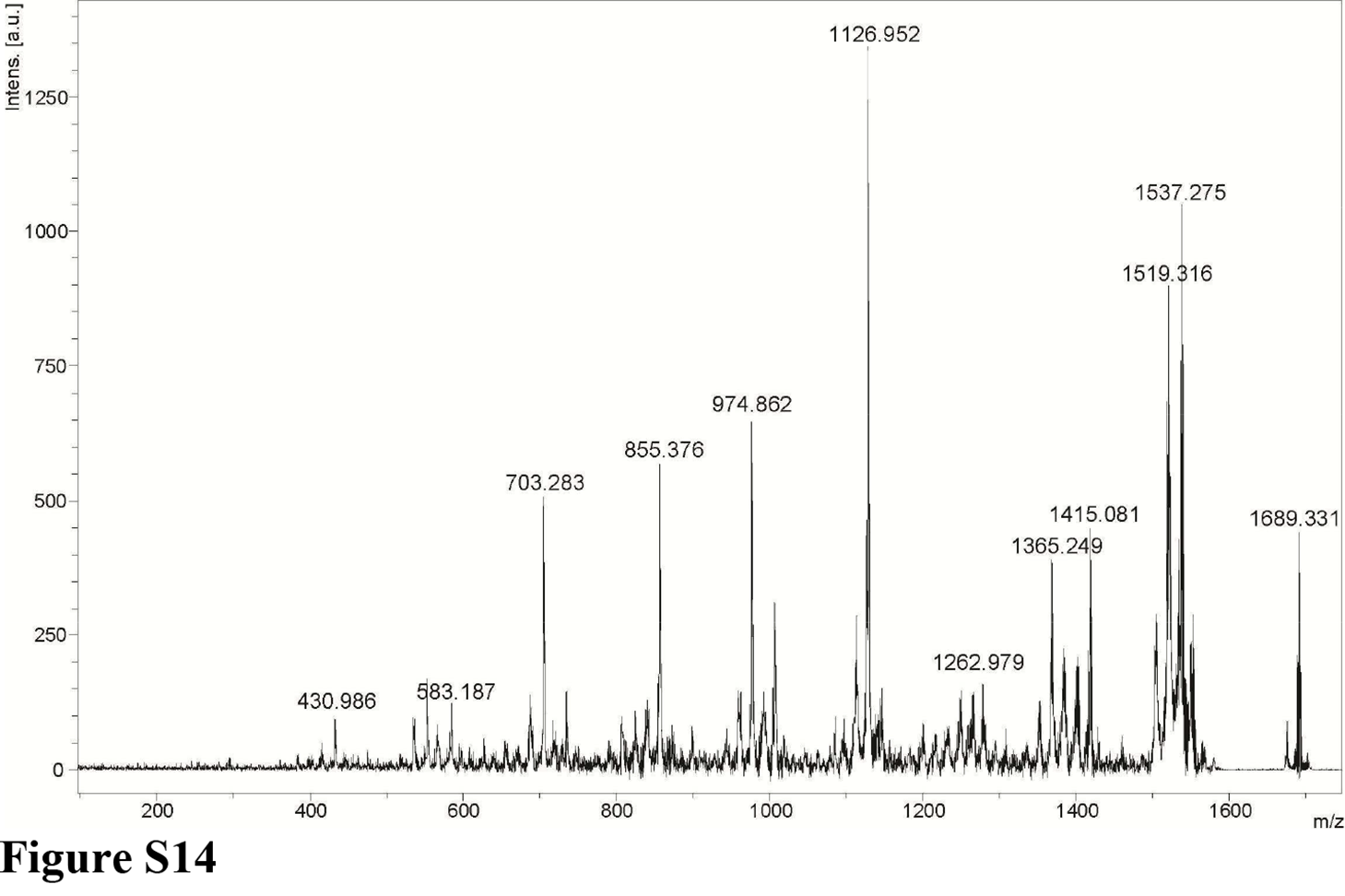

Supplement: Figure S14 — MS/MS spectrum of ion m/z 1689 from A. colubrina . (TIF) [file pone.0066257.s015.tif]

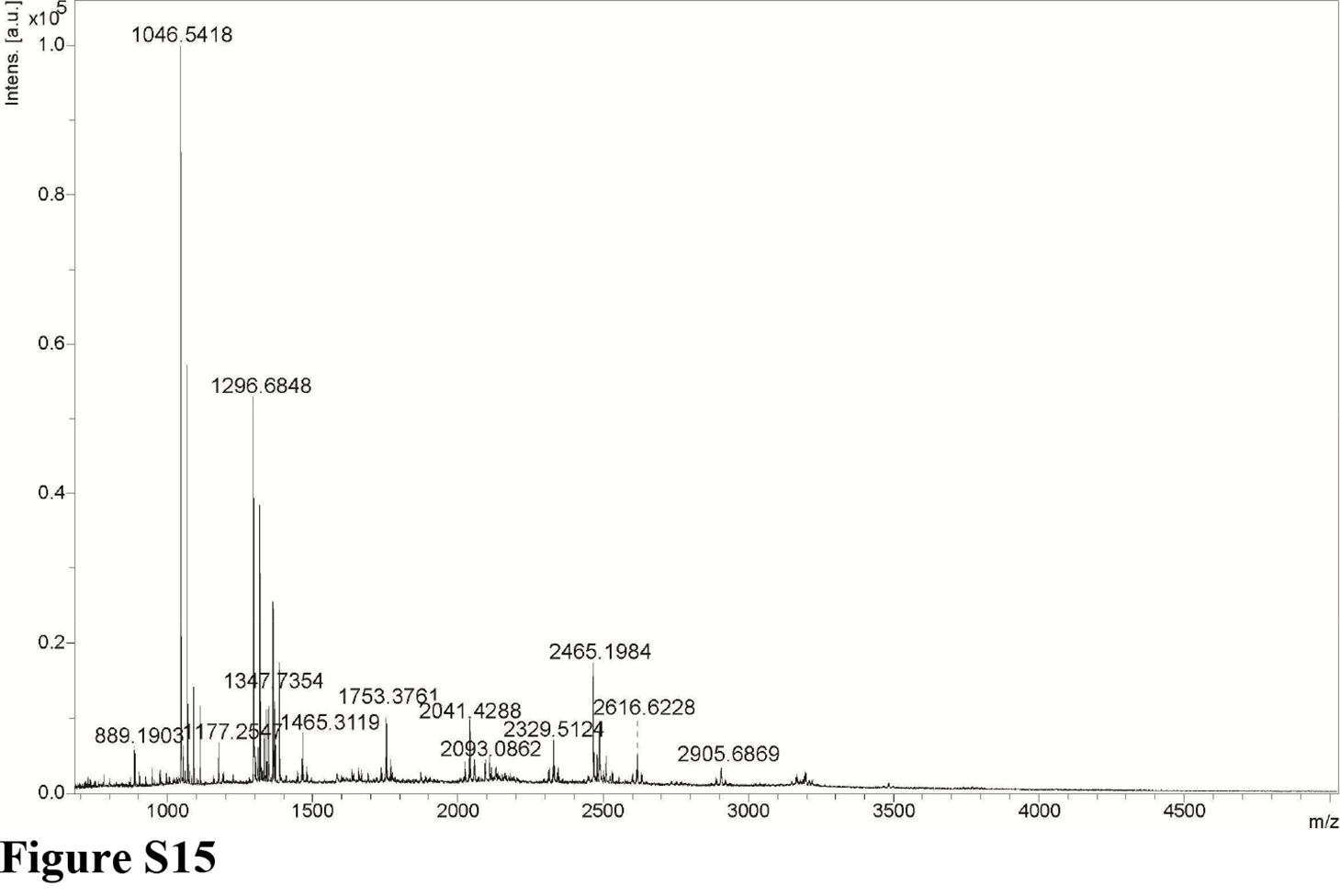

Supplement: Figure S15 — Mass spectra (positive ion mode) of the fraction obtained from C. leptophloeos with internal calibrant (peptide calibration standard II). (TIF) [file pone.0066257.s016.tif]

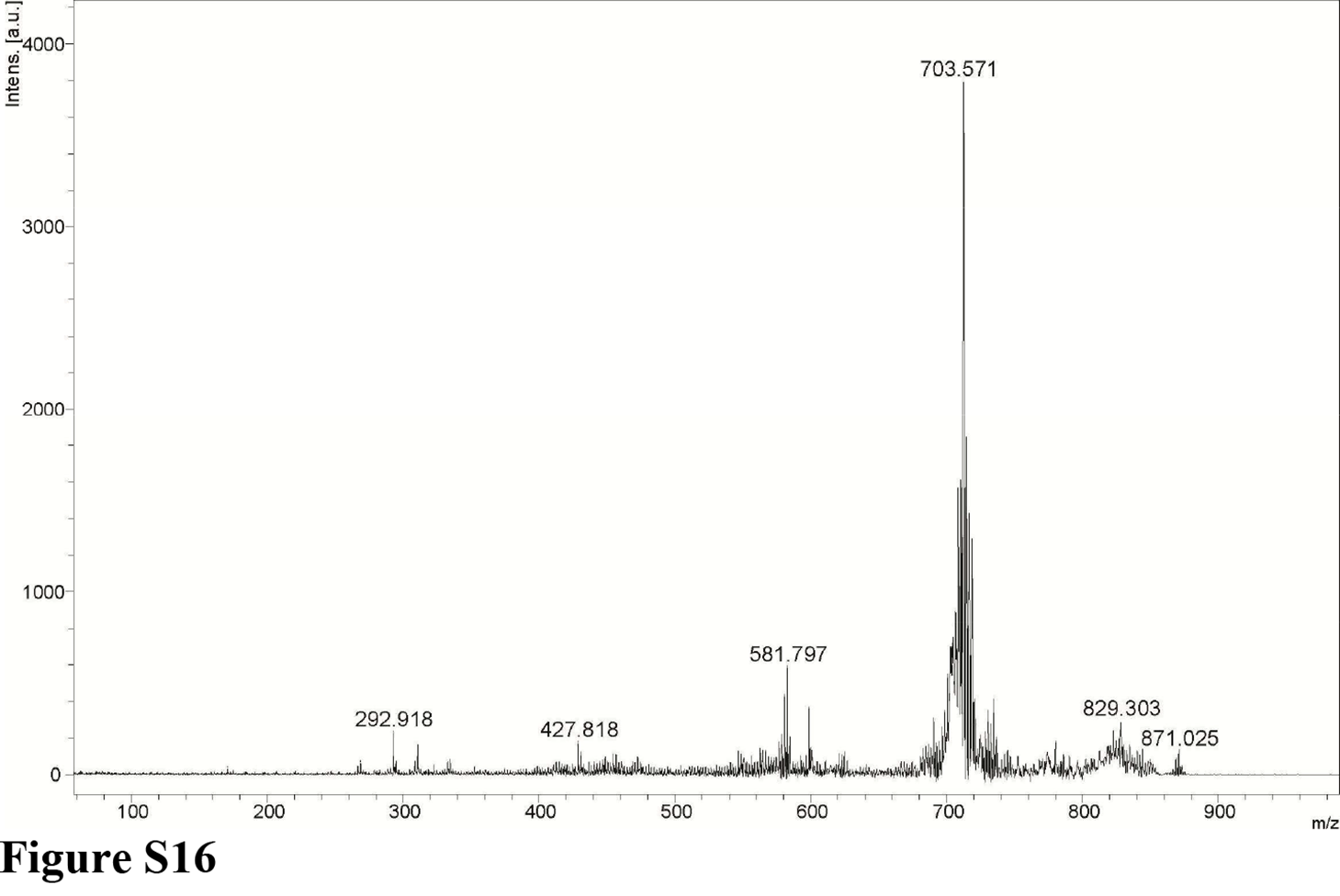

Supplement: Figure S16 — MS/MS spectrum of ion m/z 871 from C. leptophloeos . (TIF) [file pone.0066257.s017.tif]

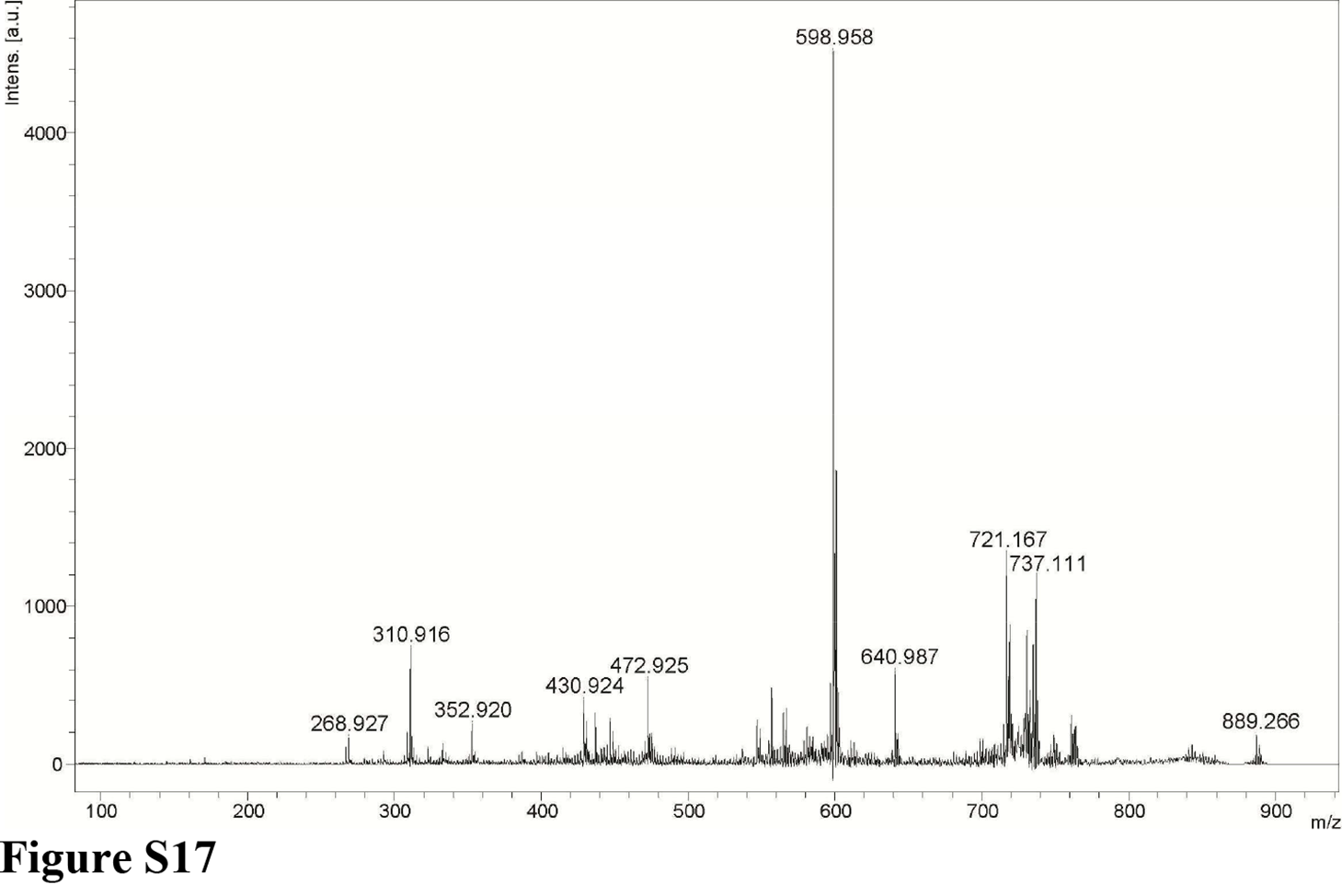

Supplement: Figure S17 — MS/MS spectrum of ion m/z 889 from C. leptophloeos . (TIF) [file pone.0066257.s018.tif]

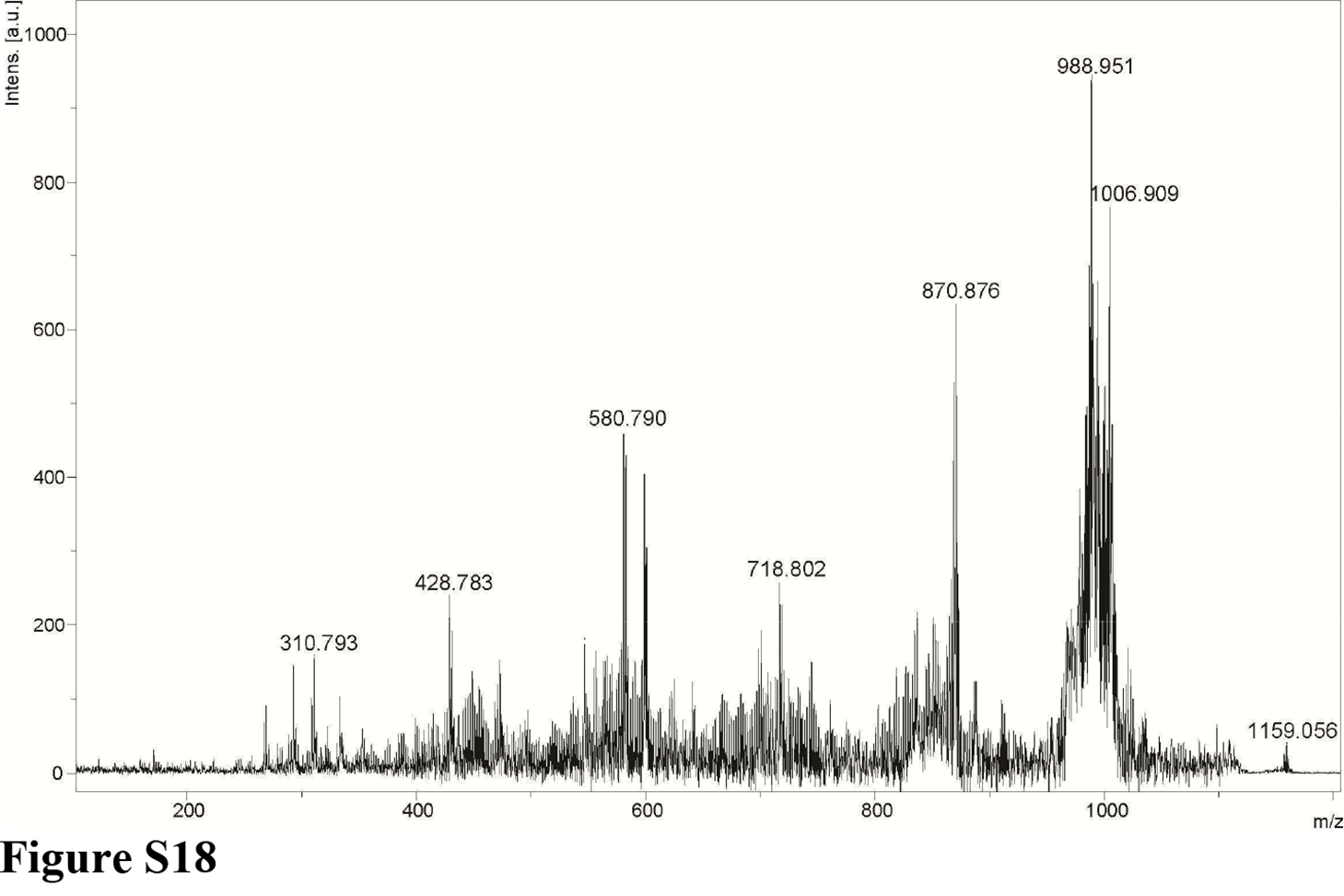

Supplement: Figure S18 — MS/MS spectrum of ion m/z 1159 from C. leptophloeos . (TIF) [file pone.0066257.s019.tif]

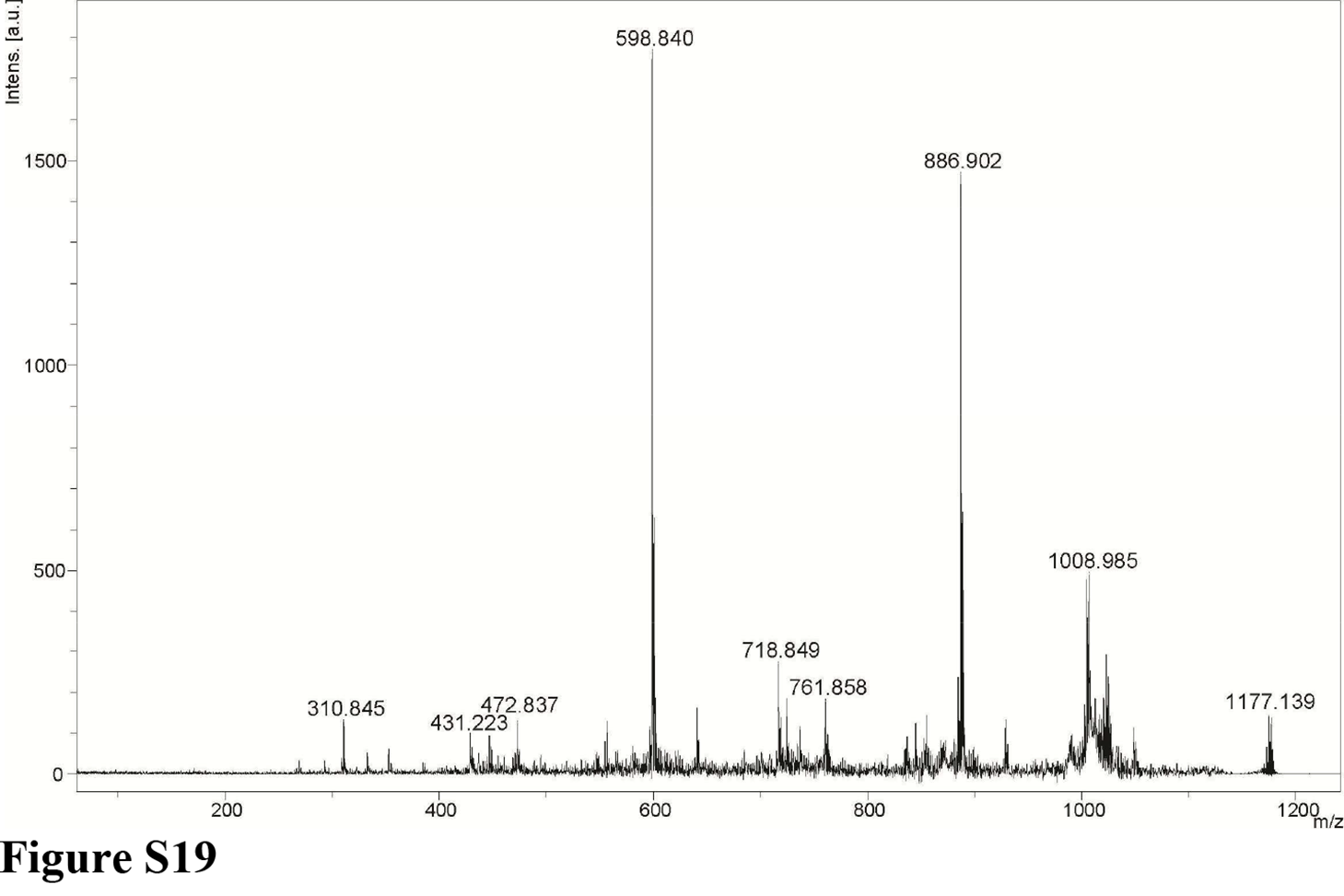

Supplement: Figure S19 — MS/MS spectrum of ion m/z 1177 from C. leptophloeos . (TIF) [file pone.0066257.s020.tif]

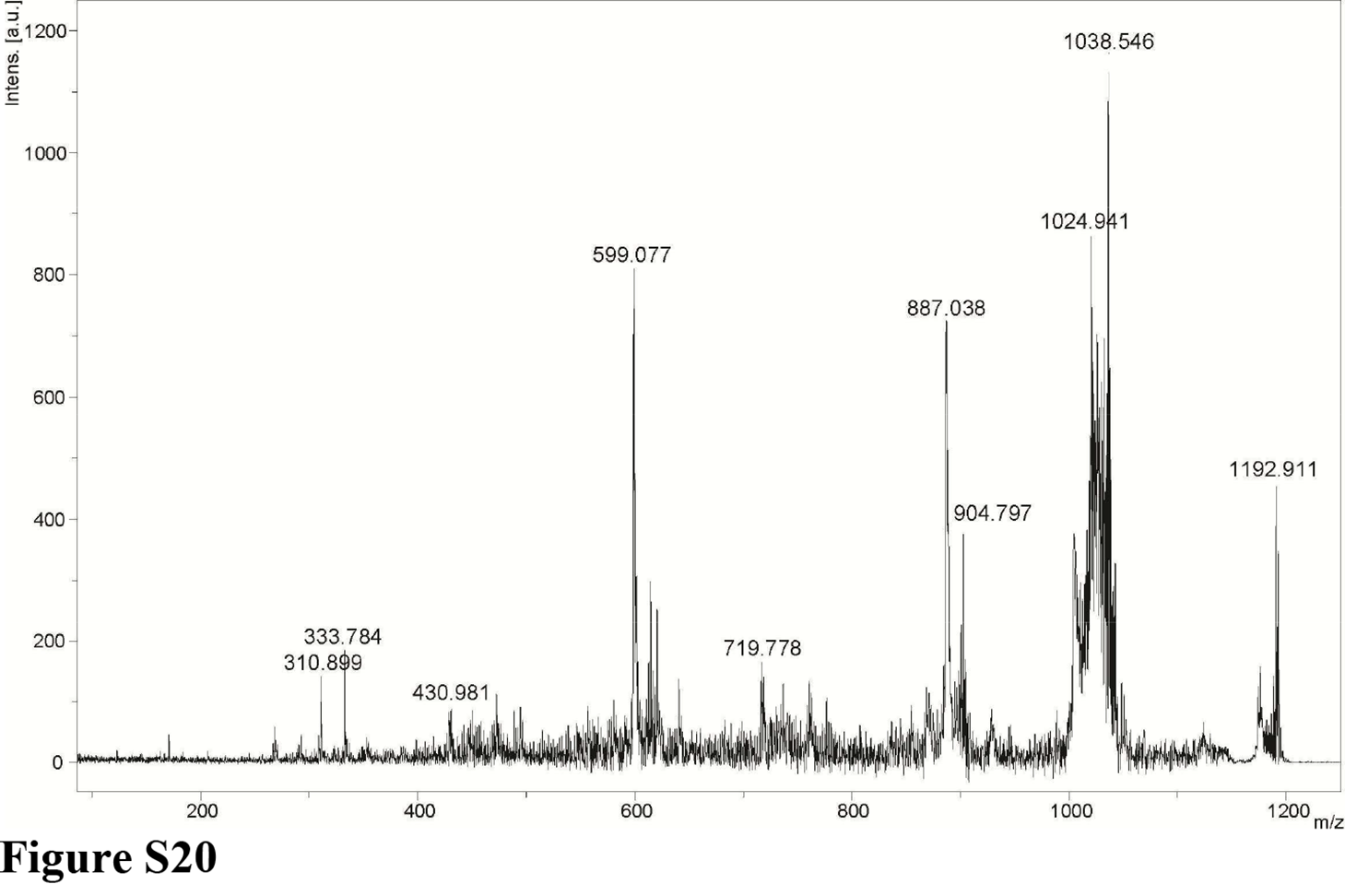

Supplement: Figure S20 — MS/MS spectrum of ion m/z 1193 from C. leptophloeos . (TIF) [file pone.0066257.s021.tif]

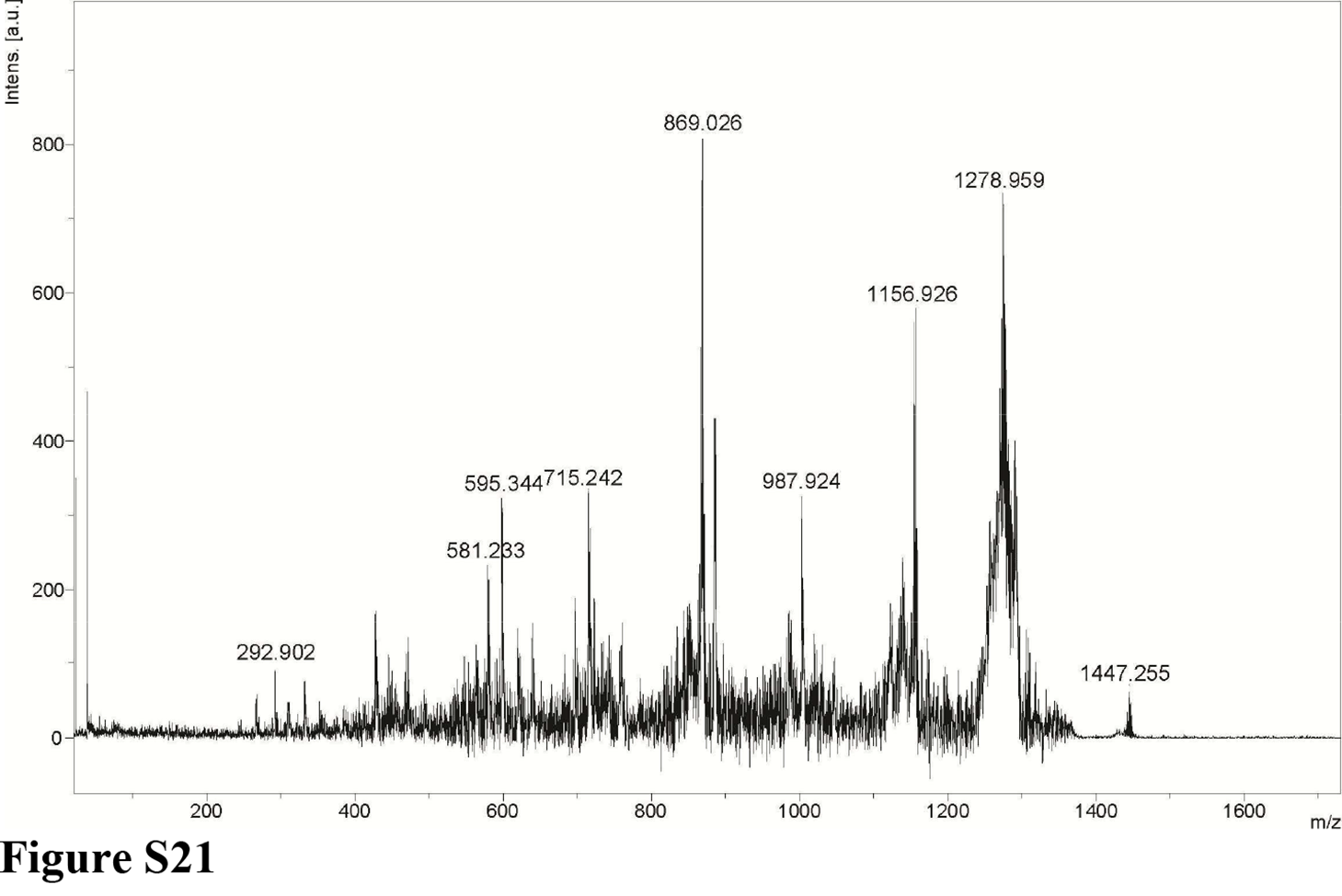

Supplement: Figure S21 — MS/MS spectrum of ion m/z 1447 from C. leptophloeos . (TIF) [file pone.0066257.s022.tif]

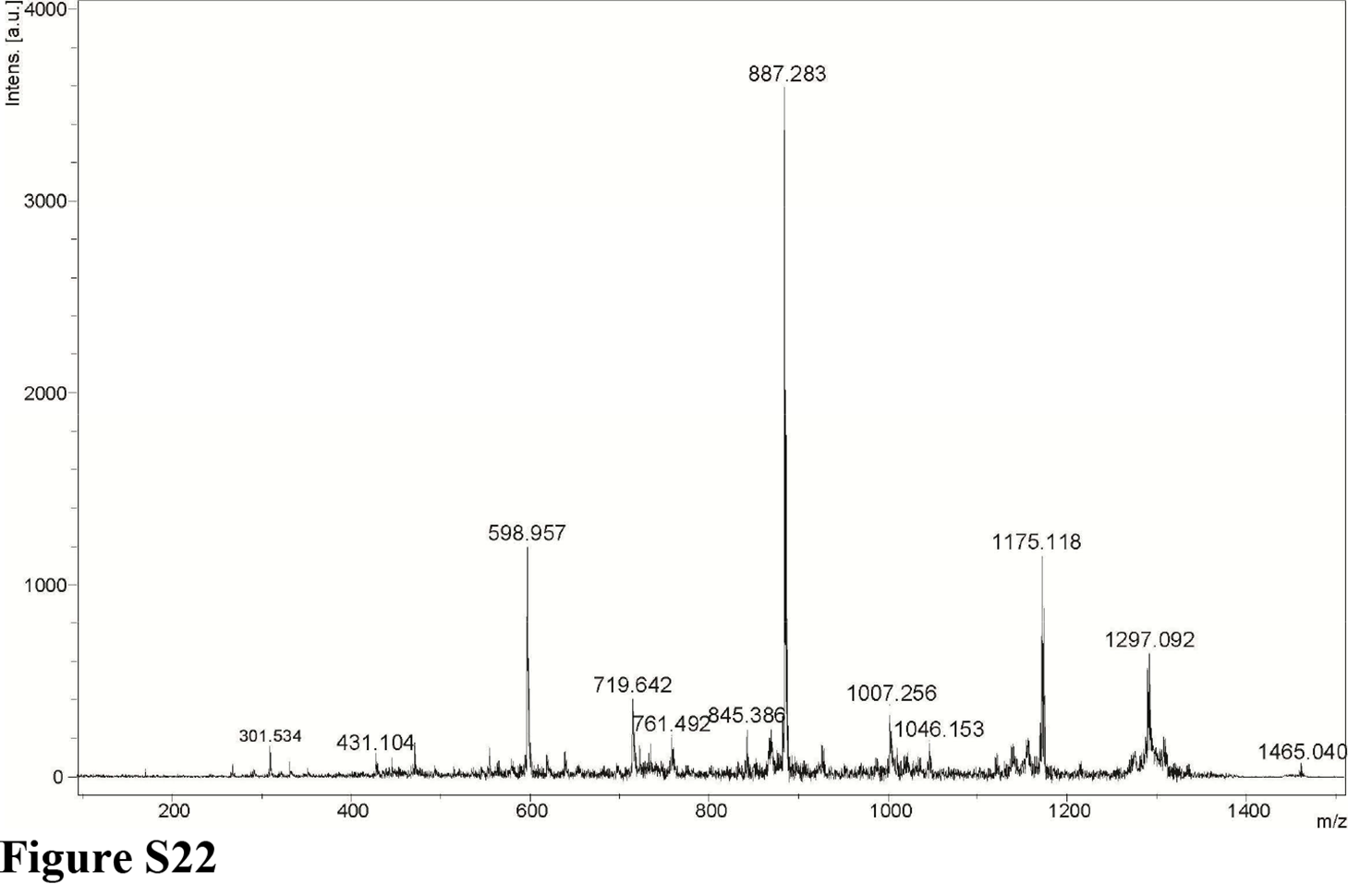

Supplement: Figure S22 — MS/MS spectrum of ion m/z 1465 from C. leptophloeos . (TIF) [file pone.0066257.s023.tif]

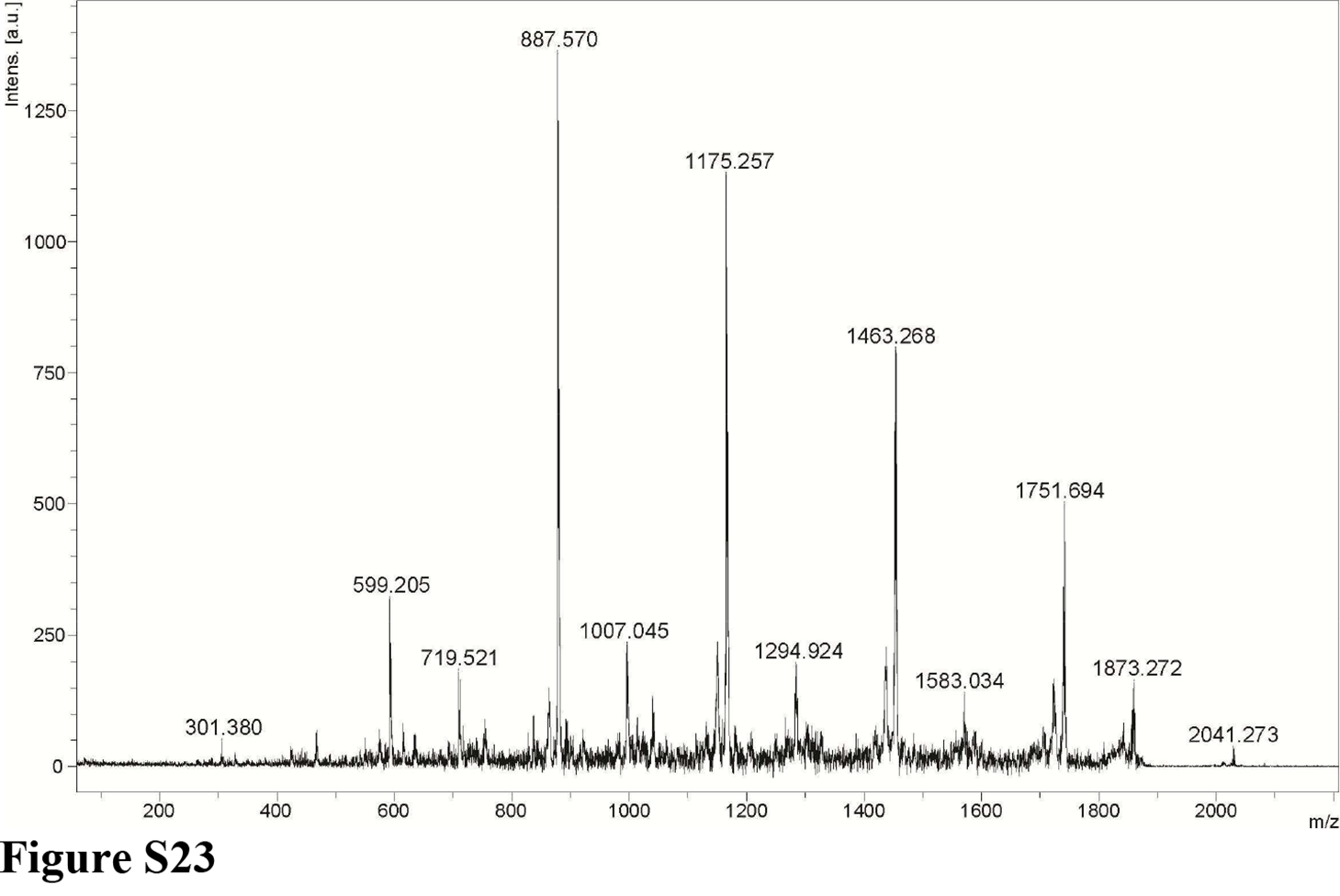

Supplement: Figure S23 — MS/MS spectrum of ion m/z 2041 from C. leptophloeos . (TIF) [file pone.0066257.s024.tif]

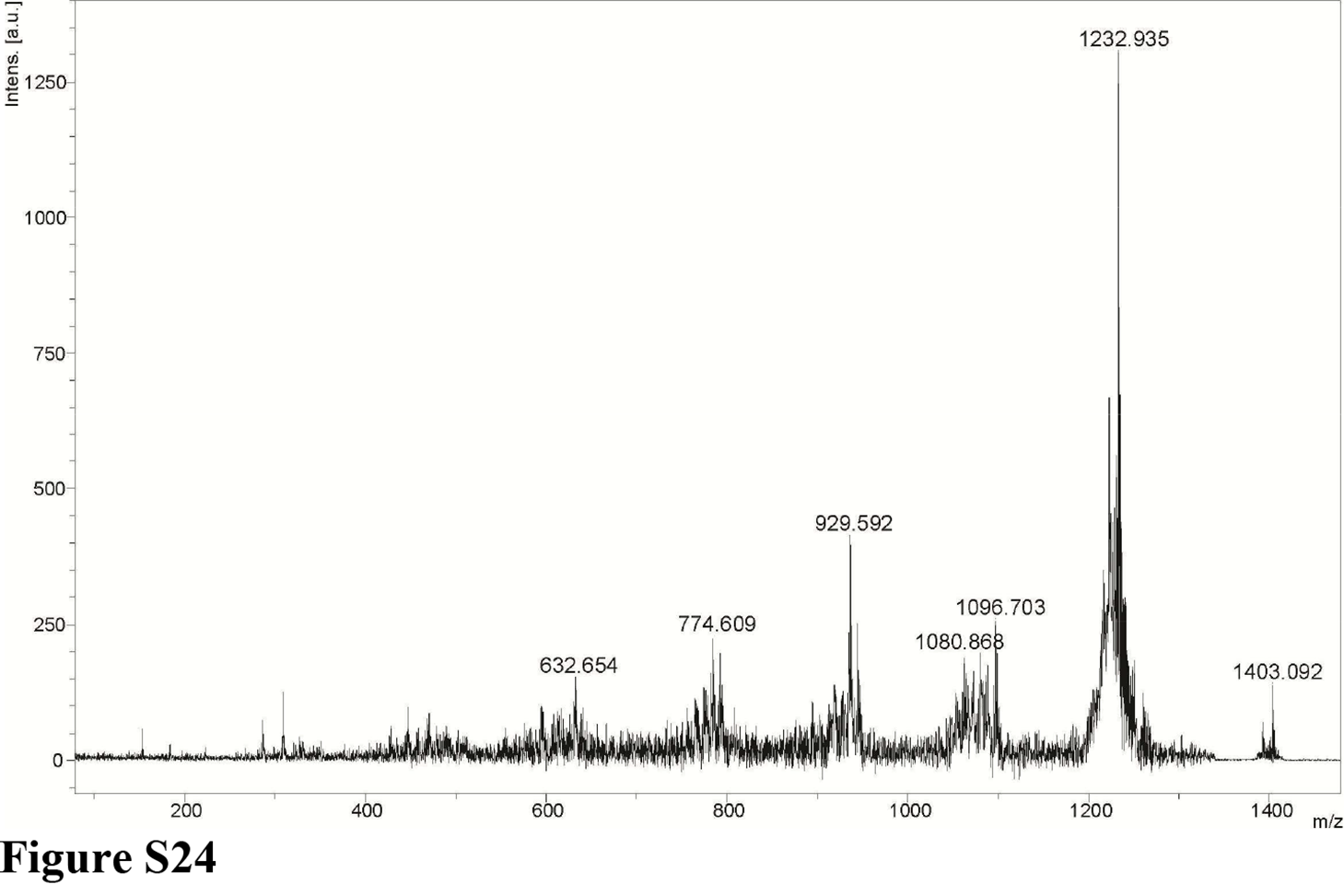

Supplement: Figure S24 — MS/MS spectrum of ion m/z 1403 from M. urundeuva . (TIF) [file pone.0066257.s025.tif]
